# Molecular approaches underlying the oogenic cycle of the scleractinian coral, *Acropora tenuis*

**DOI:** 10.1038/s41598-020-66020-x

**Published:** 2020-06-18

**Authors:** Ee Suan Tan, Ryotaro Izumi, Yuki Takeuchi, Naoko Isomura, Akihiro Takemura

**Affiliations:** 1 Graduate School of Engineering and Science, University of the Ryukyus, Nishihara, 903-0213 Japan; 2 Department of Chemistry, Biology and Marine Science, Faculty of Science, University of the Ryukyus, Nishihara, 903-0213 Japan; 3 Developmental Neurobiology Unit, Okinawa Institute of Science and Technology Graduate University (OIST), Onna, 904-0412 Japan; 4 Department of Bioresources Engineering, National Institute of Technology, Okinawa College, Nago, 905-2192 Japan

**Keywords:** Molecular biology, Reproductive biology

## Abstract

This study aimed to elucidate the physiological processes of oogenesis in *Acropora tenuis*. Genes/proteins related to oogenesis were investigated: Vasa, a germ cell marker, vitellogenin (VG), a major yolk protein precursor, and its receptor (LDLR). Coral branches were collected monthly from coral reefs around Sesoko Island (Okinawa, Japan) for histological observation by *in situ* hybridisation (ISH) of the Vasa (*AtVasa*) and Low Density Lipoprotein Receptor (*AtLDLR*) genes and immunohistochemistry (IHC) of AtVasa and AtVG. AtVasa immunoreactivity was detected in germline cells and ooplasm, whereas AtVG immunoreactivity was detected in ooplasm and putative ovarian tissues. *AtVasa* was localised in germline cells located in the retractor muscles of the mesentery, whereas *AtLDLR* was localised in the putative ovarian and mesentery tissues. *AtLDLR* was detected in coral tissues during the vitellogenic phase, whereas AtVG immunoreactivity was found in primary oocytes. Germline cells expressing *AtVasa* are present throughout the year. In conclusion, Vasa has physiological and molecular roles throughout the oogenic cycle, as it determines gonadal germline cells and ensures normal oocyte development, whereas the roles of VG and LDLR are limited to the vitellogenic stages because they act in coordination with lipoprotein transport, vitellogenin synthesis, and yolk incorporation into oocytes.

## Introduction

Approximately 70% of scleractinian corals are hermaphroditic broadcast spawners and have both male and female gonads developing within the polyp of the same colony^1^. They engage in a multispecific spawning event around the designated moon phase once a year^2–4^. Approaching the spawning season, corals undergo gametogenesis until their gametes are fully matured; oogonia appear alongside the mesoglea in each gastrodermal mesentery and differentiate into oocytes, which accumulate yolk in their cytoplasm^2,5^. Scleractinians like *Acropora* and *Montipora* have matured oocytes and sperm packaged as egg-sperm bundles^6^, which then appear at the mouth of coral polyps and are released into the seawater column a few hours after sunset. Other broadcast spawners (e.g., *Porites*, *Euphyllia*, *Pocillopora*, *Fungia*, etc) do not form egg-sperm bundles but the eggs and sperms are released directly from the mouth of their polyps. In some cases, certain coral species spawn during the day. For example, *Pocillopora meandrina*, *P. acuta* (Kaneohe Bay, Hawai’i), *P. verrucosa and P. eydouxi* (Okinawa, Japan) spawned early in the morning, while *Porites rus* (Chumbe Island, Zanzibar) and *Pavona* sp. (Gulf of Thailand) released sperms in the noon^7,8^. Although this process of oogenesis in corals is well documented^3^, there is limited information on the internal system of oogenesis in corals.

Oogenesis is a complex process involving successive mitotic and meiotic phases that are controlled by several reproductive genes and proteins. Gametes, such as egg and sperm are derived from germline cells in the gonadal region. In order to identify germline cells from other somatic cells, some germline cell specific genes (e.g. *vasa*, *piwi, nanos, oskar, gurken, aubergine* etc.) are discovered^9,10^. Among the germline cell specific genes, *vasa* and *piwi* were identified in the stony coral, *Euphyllia ancora*^11,12^. Vasa, a member of the DEAD-box RNA helicase family, acts as a translational regulator of germline-specific target mRNAs and piRNAs to determine cell outcome within germline cells^13–16^. Vasa was originally identified in *Drosophilla melanogaster*^13,17^ and afterwards, in various vertebrates (human^18^, mice^19^, chicken^20^, frog^21^, teleost^22–24^) and invertebrates (planaria^25^, sea anemone^9^, stony coral^11^, hydra^26^, and sea urchin^27^). On the other hand, Piwi is a member of the Argonaute (AGO) protein family, characterized by two major motifs, the *Piwi*/Argonaute/Zwille (PAZ) domain and the PIWI domain. Piwi proteins associate with *piwi*-interacting RNAs (piRNAs) and regulate epigenetic programming and posttranscriptional regulation, which may be involved in germline specification, germ cell maintenance, transposon silencing, and genome integrity^28–30^. In the stony coral, *E. ancora*, Vasa and Piwi are expressed in early stage germline cells and is proven to be reliable molecular markers to identify germline cells in corals^11,12^. It was also reported that Vasa and Piwi in marine invertebrates (stony coral^11,12^, sea anemone^9^, jellyfish^31^, and hydroids^32^) acts as a maternal factor in formation and determination of germline and multipotent stem cells during embryogenesis. Furthermore, both Vasa and Piwi plays a crucial role in germ cell formation, maintenance, and development, as well as gamete maturation^9,32–38^. Post-transcriptional studies showed that a null mutation removing the entire Vasa coding region interfered with reproductive capabilities resulting in female sterility with severe defects, including abnormal germline differentiation and oocyte determination^34–38^. In *D. melanogaster*^34,37^ and *C. elegans*^35^, defects in germline proliferation and abnormal/absent of oocytes in the gonad were observed when Vasa gene was neutralized by RNAi. In addition, delayed spermatogenesis and defective sperm was observed in *C. elegans*^35^. While in zebrafish^36^ and mice^38^, mutation in Vasa genes hinders the progress of spermatogenesis and defects in germ cell proliferation and differentiation. As germline cells ensure the continuous production of gametes, germline-specific genes (for example, Vasa) that are expressed in germline cells may have a significant role in gametogenesis in the reproductive cycle of scleractinian corals.

Vitellogenesis is the process of vitellogenin (VG) synthesis and incorporation into developing oocytes. In most animals, VG is synthesised in the somatic tissues (liver in vertebrates^39^; fat body in insects^40^; hepatopancreas in crustaceans^41,42^; intestine in sea urchins^43^ and in ovary of many other animals^44^) and transported to the gonad to be accumulated in the oocytes^45^. On the other hand, VG of corals^46^ and sea anemone^47,48^ is synthesised within putative ovarian tissues by mesenterial somatic cells in the vicinity of the oocyte. In the sea anemone, yolk synthesis involves in both autosynthetic and heterosynthetic processes including the biosynthetic activity of the Golgi complex and the uptake of VG via endocytosis^47,48^. Ultrastructural studies of the sea anemone^47,48^ and jellyfish^49^ revealed the presence of specialized gastrodermal cells (known as “trophocytes” and microvilli around the oocytes), which play an important role in nutrients transportation during vitellogenesis. There is also evidence that extraoocytic uptake of VG is manifested by the proliferation of endocytotic pits and vesicles over the surface of the oolemma in sea anemone^47^. The VG uptake process in corals has yet to be elucidated, although two possibilities have been proposed: either via transmesoglea pores or the somatic cell-oocyte contact through transmesoglea pores and would be subsequently taken up by oocytes through receptor-mediated endocytosis^50^. Due to the large size of the VG protein, the latter possibility is more likely. VGR belongs to the supergene family of low-density lipoprotein receptor (LDLR)-related proteins that have co-evolved in egg-laying and viviparous animals^51^. Regarding VGR studies in vertebrates, proteins with a high affinity for VG have been identified and characterised on the oocyte membrane from chicken^51–53^, frog^53^, and several fish species, including the rainbow trout^54,55^ and the sea bass^56^. On the other hand, VGR and LDLR have been identified in invertebrates such as insects^57^, mites^58^, ticks^59,60^, shrimps^61–63^ and crabs^64^. These findings showed that the VGR transcript was up-regulated during the initial phase of VG uptake into the oocyte, indicating that VGR is critical for yolk protein absorption and ovary maturation. Although VGR/LDLR has yet to be identified in corals, it is probable that VG is synthesised in the somatic cells of ovarian tissues and incorporated into oocytes via receptors during vitellogenesis.

The aim of this study was to clarify the oocyte development of the scleractinian coral *Acropora tenuis*^65^, a hermaphroditic branching stony coral belonging to the Acroporidae family, from the molecular point of view. The genome database of its sister species, *A. digitifera*^66^, was established in the recent years, making it easier to obtain desired gene information. Most importantly, although there is vast information of *A. tenuis* on its spawning pattern, planulae production and fertilization studies^4^, there are no molecular and cellular studies of this species. We isolated and characterised the *vasa* and *LDLR* genes from *A. tenuis*, and to examine their molecular and cellular roles in oocyte development. We determined the localisation of *AtVasa* and *AtLDLR* mRNA expression using *in situ* hybridisation (ISH) and the immunoreactivity against AtVasa and vitellogenin (AtVG) using immunohistochemistry (IHC). In addition, we investigated the relationship between these gene and protein profiles and oocyte development during the annual reproductive cycle of this species. This study holds great significant as it helps us to understand the flow of oogenesis in Acroporid corals, as well as the physiological and molecular role of the reproduction related genes (*Atvasa* and *AtLDLR*) and proteins (Atvasa and AtVG) throughout the oogenesis process. This study will be the first to show molecular evidence on the involvement of LDLR during vitellogenesis in corals.

The results of this study will be of great benefit to understand the physiological process underlying oogenesis in scleractinian corals as it would provide as; (1) Benchmark for reproductive studies of other species of scleractinian corals, (2) Molecular indicators to identify the internal factors that promote oogenesis in scleractinian corals, (3) Stepping stone for future coral reproductive studies such as endocrine/neuroendocrine pathways, effect of environmental factors on gametogenesis/spawning and coral culture experiment under artificial conditions, (4) New insights of reproductive mechanisms in other cnidarians.

## Results

### Gamete development of *A. tenuis* and classification of development stages

**Figure 1:**
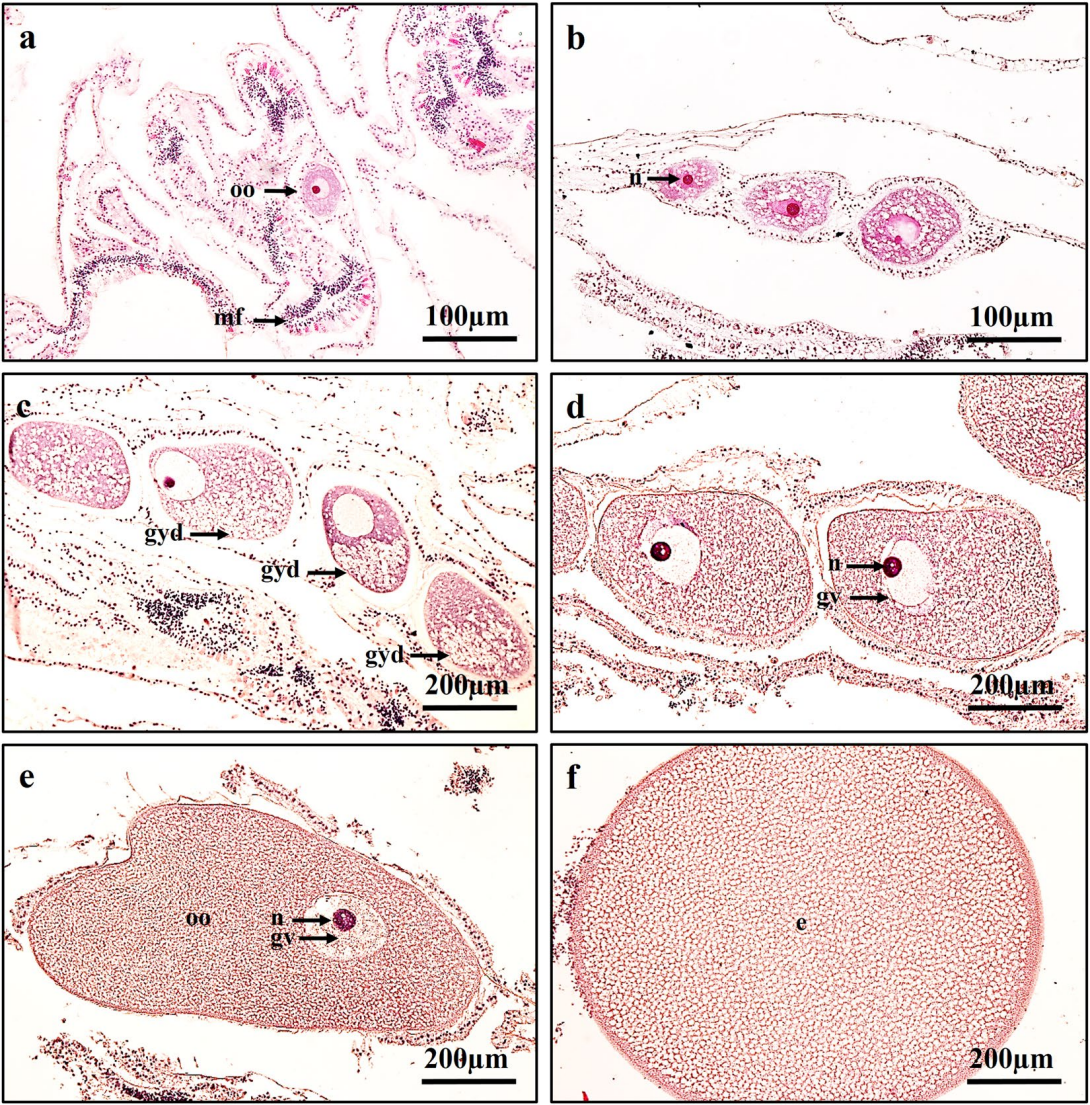
Distinct oocyte stages of *A. tenuis*. (**a**) Stage I; oocyte enveloped in mesoglea, showing nucleus. (**b**) Stage II; oocyte, cytoplasm, and nucleus have grown in size. (**c**) Stage III; oocyte with finely granular yolk/cytoplasm. Yolk polarity and migration of nucleolus to periphery of nucleus observed. (**d,e**) Stage IV-V; oocyte grow tremendously in size. (**f**) Stage VI; Final stage (Egg). oo; oocyte, mf; mesentery filament, n; nucleus, gv; germinal vesicle, gyd; granular yolk disposition, e; egg.

**Figure 2:**
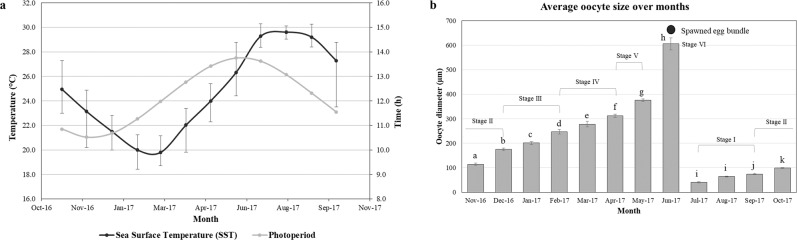
(**a**) Annual changes in average sea surface temperature and photoperiod in Sesoko station, Okinawa, Japan. Black line indicates Sea Surface Temperature (SST) and grey line indicates Photoperiod. Error bar indicates maximum and minimum value of SST. (**b**) Monthly changes in mean diameter of oocytes of *A. tenuis*. The data are presented as mean ± SE. Significant differences (P < 0.05) are indicated with the different lower case letters. Respective oocyte stages are shown above each bar graphs.

Histological observations revealed that the site of gametogenesis (in the gonad) was located within the mesoglea of the mesentery, which was located between the mesentery filament and the retractor muscles in the endoderm (Fig. 1). One polyp may hold up to two pairs of testes and two pairs of ovaries. Each filament contained between 3 and 10 clusters of oocytes/spermatocytes. When oocytes were compared between the colonies collected at each sampling time, the gonads were found to develop synchronously toward the date of spawning. The collection of coral branches began in November 2016. The histological observations revealed that, after the initial collection date, the oocytes increased in size (diameter) and grew steadily over the months until mass spawning occurred in June 2017 (Figs. 1a–f,2b). Immature oocytes were first observed in histological sections collected from colonies in July 2017 (Figs. 1a, 2b).

### Seasonal changes in oogenesis

Annual changes in environmental factors (sea surface temperature [SST] and photoperiod) and oocyte diameters of *A. tenuis* are shown in Fig. 2. During the sample collection period, SST decreased from November (25.2 °C) to March (20.3 °C, which was the lowest temperature observed). SST steadily increased from April (22.6 °C) to August (30.1 °C), and began decreasing in September (29.7 °C). Conversely, the shortest photoperiod was observed in December (10.31 h). It gradually increased to its maximum in June (13.45 h) and decreased steadily thereafter (Fig. 2a).

Early vitellogenic oocytes were observed at Stage II of oogenesis in November (114.33 ± 4.51 µm) and December (175.33 ± 5.38 µm), which then continued to develop into Stage III in January (201.27 ± 5.73 µm) and February (247.26 ± 9.38 µm). The mean diameter of vitellogenic oocytes (Stage IV and V) was 278.19 ± 11.50 µm in March, which increased in April (312.44 ± 6.54 µm) and peaked in May (376.19 ± 5.28 µm) before the release of fully matured eggs (Stage VI) in June (605.53 ± 24.04 µm). This value significantly decreased in July (41.27 ± 2.70 µm). The beginning of Stage I of oogenesis was observed from July to September (74.52 ± 1.92 µm) (Fig. 2b).

### Cloning and characteristics of *AtVasa*, *AtLDLR* and *AtVG*

The cDNAs of *AtVasa*, *AtLDLR* and *AtVG* were partially cloned in the present study. The cDNA of *AtVasa* had an open reading frame (ORF) composed of 1242 bp (413 amino acid residues). It contained conserved domains that were also found in the ORFs of other cnidarians and invertebrates (Supplementary Fig. S1): the Q-motif (XXXXPTPXQ), ATPase motifs (AXXGXGKT and DEAD), and motifs involved in ATP binding and cleavage (PTREL, GG, and TPGRL). Phylogenetic analyses revealed that AtVasa exclusively clustered with Vasa proteins of other cnidarian species (Supplementary Fig. S2). Conversely, the cDNA of *AtLDLR* had an ORF composed of 1263 bp (420 amino acid residues). Based on the deduced amino acid sequences, the cDNAs were identified as members of the LDLR superfamily. The sequence contained LDLR Domain Class A, the DXSDE motif, the FWTD motif, cysteine-rich repeats, and the following conserved modules: a calcium binding site, a putative binding site, and epidermal growth factors, which were also found in human and other cnidarian ORFs (Supplementary Fig. S3). Phylogenetic analyses revealed that AtLDLR exclusively clustered with VGR/LDLRs of other cnidarians and invertebrates (Supplementary Fig. S4). The cDNA of *AtVG* had an open reading frame ORF composed of 1458 bp (484 amino acid residues). The sequence contained a domain of unknown function (DUF1943) and cleavage sites of subtilisin family endoproteases, which were also found in other scleractinian corals (Supplementary Fig. S5). Phylogenetic analyses revealed that *AtVG* exclusively clustered with VGs of other cnidarian ORFs (Supplementary Fig. S6).

### Expression of *AtVasa*, *AtLDLR* and *AtVG* in tissues

*AtVasa* and *AtLDLR* were expressed in all branches surveyed in the present study. *AtVG* was expressed in matured branch but not in eggs. No amplified products were detected in the negative control. The expression of *β-actin* mRNA was used as positive control and detected in all branches (Supplementary Figs. S7–S9).

**Figure 3:**
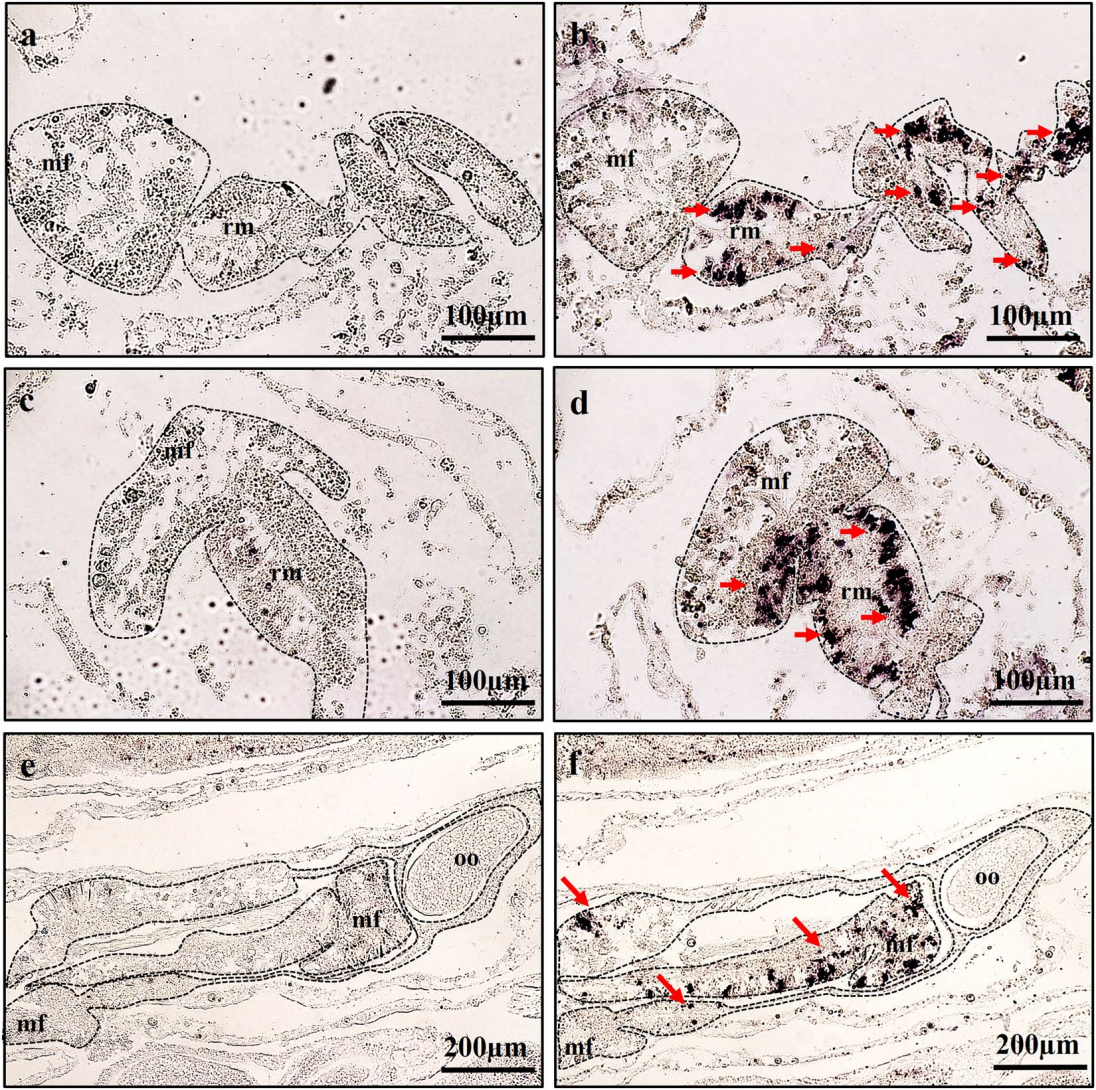
Expression profiles of *AtVasa* transcript in mesentery tissues. (**a–f**) Localization of *AtVAsa* mRNA-positive cells in the retractor muscles (rm) area of mesentery tissues. (**a,c,e**) showed section stained with *AtVasa* sense probe. (**b,d,f**) showed section stained with *AtVasa* anti-sense probe. Purple colouration (ALP reaction) indicates *AtVasa* transcript signals. mf; mesenterial filament, oo; oocyte.

**Figure 4:**
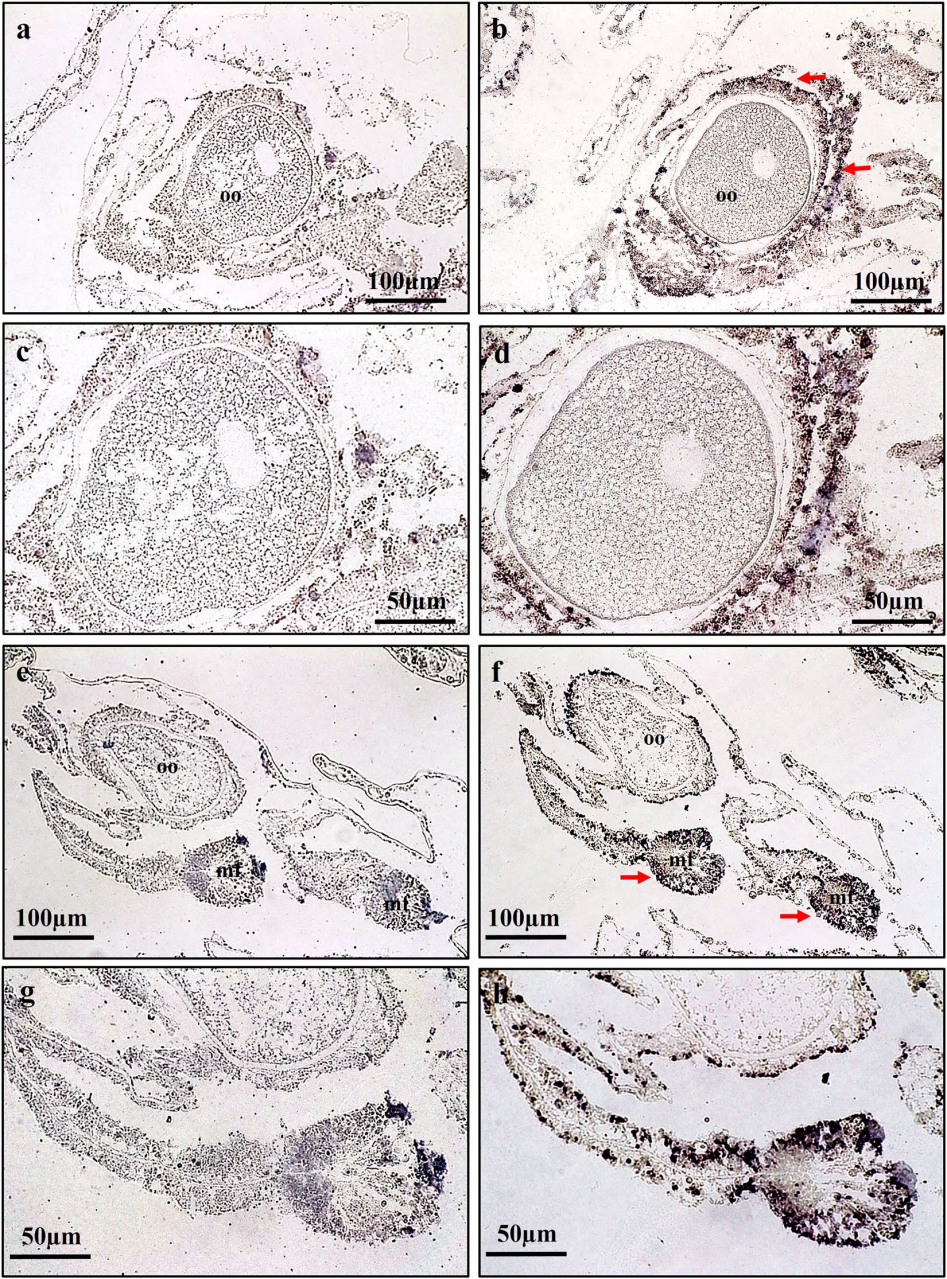
Expression profiles of *AtLDLR* transcript in putative ovarian and mesentery tissues. (**a–h**) Localization of *AtLDLR* mRNA-positive cells in putative ovarian and mesentery tissue. (**a,c,e,g**) showed section stained with *AtLDLR* sense probe. (**b,d,f,h**) showed section stained with *AtLDLR* anti-sense probe. Purple colouration (ALP reaction) indicates *AtLDLR* transcript signals. (**c,d,g,h**) showed higher magnification views of (**a,b,e,f**) respectively. mf; mesenterial filament, oo; oocyte.

The localisation of *AtVasa* and *AtLDLR* mRNA expression in coral tissues was investigated using ISH (Figs. 3, 4; Supplementary Fig. S10, S11). *AtVasa* transcripts were observed in the putative germ cells (male or female) and distributed throughout the gonadal regions in the retractor muscles of the mesentery along the mesoglea (Fig. 3b,d,f). The germline cells maintained their undifferentiated state and remained in the specific site of mesenteries. These specific signals were not observed when the sense probe was applied (Fig. 3a,c,e).

The *AtLDLR* transcript was detected in the reproductive tissues of *A. tenuis* when the coral tissues were hybridised with the anti-sense probe; it was located in the tissues of the mesentery, which are the putative ovarian tissues surrounding the oocyte, and in close proximity to the oocytes (Fig. 4b,d). Additionally, this transcript was also found in the mesoglea and mesenterial filaments (Fig. 4f,h). These specific signals were not observed when the sense probe was applied (Fig. 4a,c,e,g).

### Detection of immunoreactivity of AtVasa and AtVG in tissues

**Figure 5:**
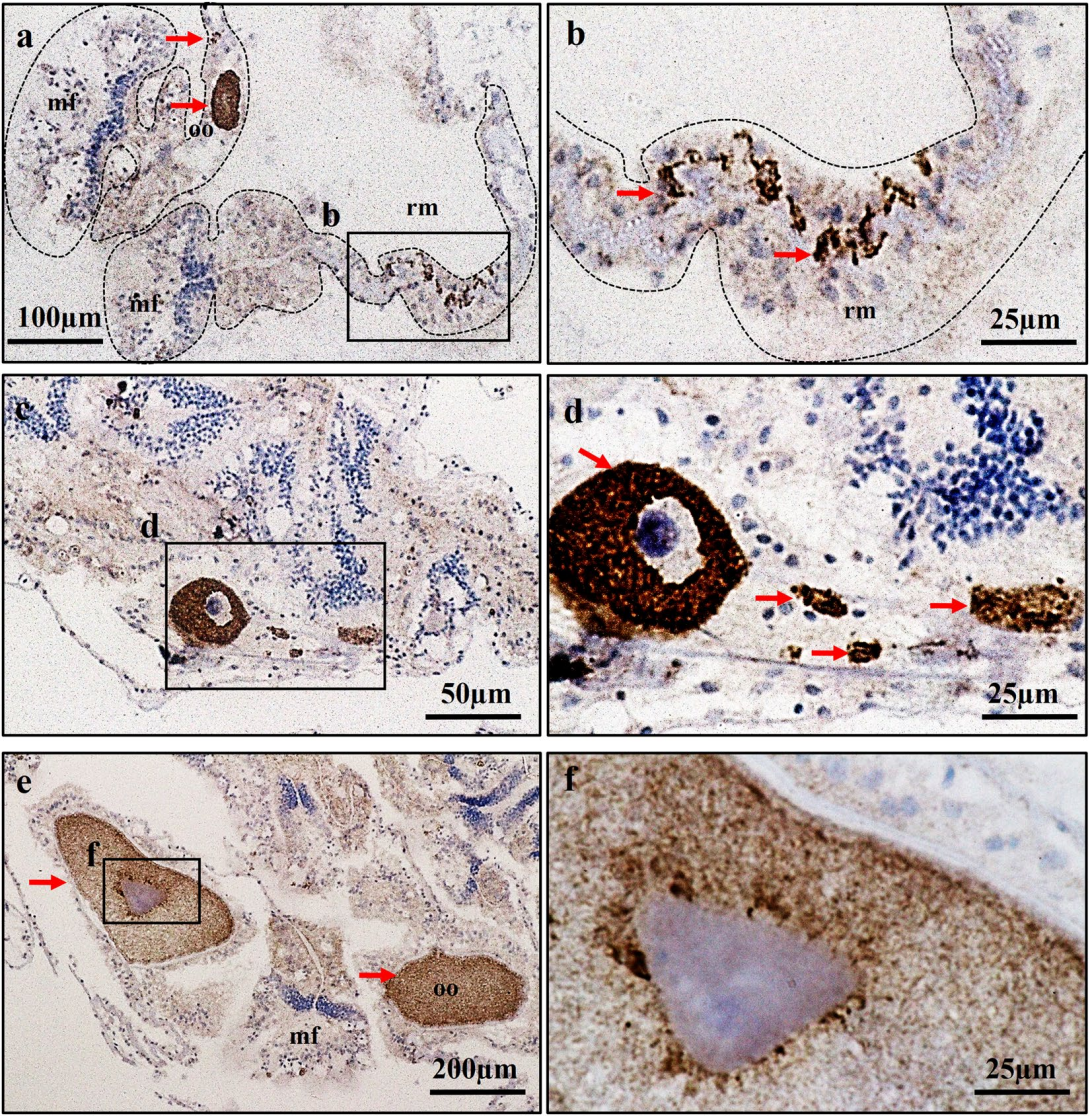
Characterization of Vasa immunoreactivity (AtVasa) in *A. tenuis* tissues. (**a**) AtVasa positive cells located in germ cells in the retractor muscle (rm); (**b**) A higher magnification view of the inset shown in (**a**). (**c**) AtVasa positive early oocyte and germ cells located along the meseoglea; (**d**) A higher magnification view of inset shown in (**c**). (**e**) AtVasa positive in oocyte cytoplasm; (**f**) A higher magnification view of inset shown in (**e**) Arrow indicates AtVasa immunoreactivity signals. mf; mesenterial filament, oo; oocyte.

**Figure 6:**
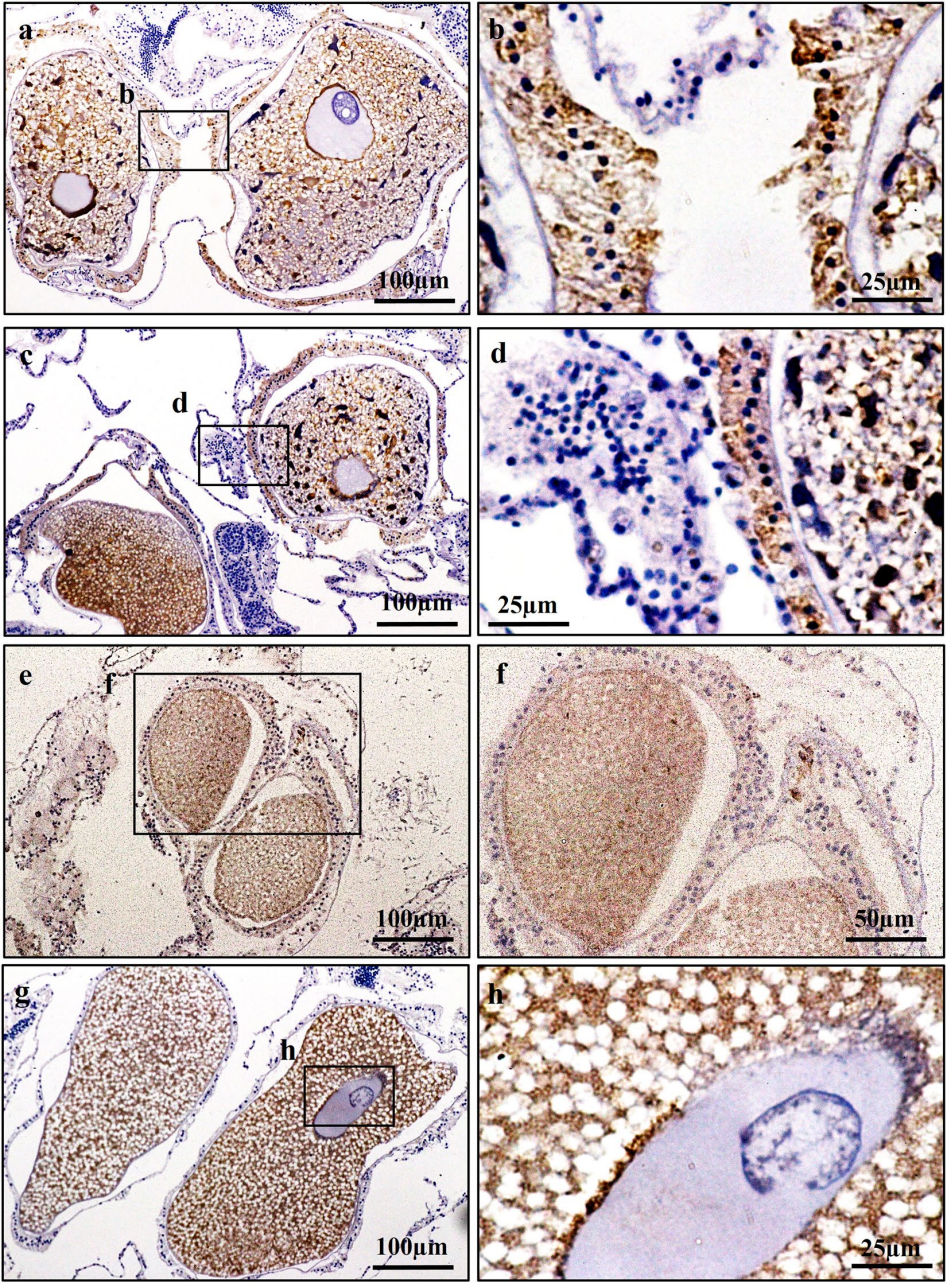
Characterization of VG immunoreactivity (AtVG) in *A. tenuis* tissues. (**a,c**) AtVG positive signal located in putative ovarian tissues; (**b,d**) A higher magnification view of the inset shown in (**a,c)** respectively; (**e,g**) AtVG positive in oocyte cytoplasm; (**f,h**) A higher magnification view of inset shown in (**e,g**) respectively. Arrow indicates AtVG immunoreactivity signals.

IHC was used to determine the localisation of immunoreactivity of AtVasa and AtVG in the tissues of *A. tenuis* (Figs. 5,6). AtVasa positive signals were observed in the possible germline cells (male or female) that were located in the retractor muscles of the mesentery (Fig. 5a,b), and in the cytoplasm of early oocytes (Fig. 5c,d), and oocytes (Fig. 5e,f). When the coral tissues were incubated with anti-EaVg, immunoreactivity against AtVG was detected in the mesenterial somatic cells (putative ovarian tissues) (Fig. 6a,b,c,d), and oocytes of *A. tenuis* (Fig. 6e,f,g,h).

### Changes in tissue transcript and protein levels of Vasa with gametogenesis

**Figure 7:**
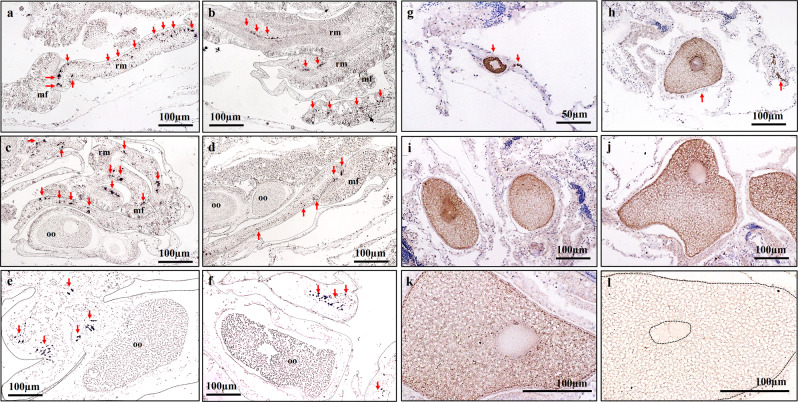
The presence of mRNA expression *of AtVasa* in germ cells **(a–f)** and immunoreactivity of AtVasa (**g–l**) in *A. tenuis* oocytes at different development stages of oogenesis. ISH: (**a,b**) Stage I-II; (**c,d**) Stage III; (**d,e**) Stage IV-V. IHC: (**g**) Stage I; (**h**) Stage II; (**i**) Stage III; (**j,k**) Stage IV; (**l**) Stage V. Arrow indicate *AtVasa* transcript and AtVasa immunoreactivity. rm; retractor muscle, mf; mesentery filament, oo; oocyte.

The relationship between *AtVasa* or AtVasa and oocyte development was analysed using ISH and IHC of *A. tenuis* tissues at different stages. *AtVasa* transcripts were observed via ISH in the germ cells throughout the retractor muscles of the mesentery from Stage I to Stage V (Fig. 7a–f; Supplementary Fig. S12). Although *AtVasa* mRNA expression was observed in the nuclei of germline cells, no *AtVasa* signal was observed in the cytoplasm of oocytes (Fig. 7c,d,e,f). Immunoreactivity against AtVasa was observed in the cytoplasm of oocytes via IHC (Fig. 7g-l), and the signal became weaker as the oocytes developed. The strongest signal was seen in the germ cells and in the earlier stages of oocyte at Stage I (Fig. 7g). The signals became weaker from Stage II to Stage V (Fig. 7h–l), during which the signal was faintly detected (Fig. 7l).

### Changes in tissue AtLDLR mRNA expression and AtVG immunoreactivity with gametogenesis

**Figure 8:**
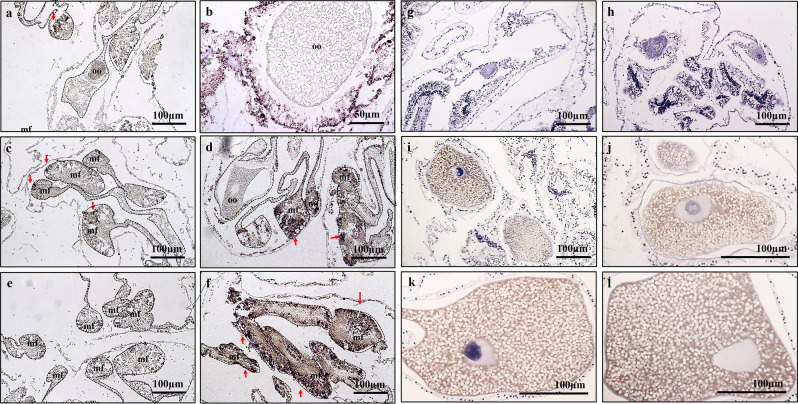
Comparison of mRNA expression of *AtLDLR* during non-vitellogenic phase, Stage I & II (**a,c,e**) and vitellogenic phase, Stage III-V **(b,d,f)** of *A. tenuis*. Meanwhile. (**g-l**) shows the seasonal change of AtVG immunoreactivity at different development stages of oogenesis. IHC: (**g)**. Stage I; (**h**) Stage II; (**i–j**) Stage III; (**k**) Stage IV; (**l**) Stage V. oo; oocyte. Arrow indicate *AtLDLR* transcript.h.

The relationship between *AtLDLR* expression and oocyte development was analysed using ISH of *A. tenuis* tissues at different stages. Tissues (putative ovarian tissues in the mesentery and mesentery filaments) and oocytes from *A. tenuis* were observed, and positive signals were strongly detected at Stages III, IV and V (the vitellogenic phase), but weak/no signal at Stages I and II (the non-vitellogenic phase) (Fig. 8a–f; Supplementary Fig. S13).

The relationship between immunoreactivity against AtVG and oocyte development was analysed using IHC of *A. tenuis* tissues at different stages. Tissues (putative ovarian tissues in the mesentery and mesentery filament) and oocytes from *A. tenuis* were observed, and immunoreactivity against AtVG was faintly detected in the cytoplasm of oocytes or not detectable at Stages I (Fig. 8g) and II (Fig. 8h). The signals became stronger as oocytes developed from Stage III to IV, and became strongest at Stage V (Fig. 8i–l).

## Discussion

The cDNAs of *AtVasa*, *AtLDLR* and *AtVG* were successfully cloned and its respective proteins were characterized. The alignment of the deduced amino acid sequences and phylogenetic analyses suggest that AtVasa belongs to the *vasa* DEAD box helicase family^9,11,26,27,32,33^, AtLDLR belongs to the LDLR superfamily^51–64,67^ and AtVG belongs to the large lipid transfer protein superfamily^46,68,69^. Through partial cloning of *AtVasa*, seven out of nine conserved domains characteristic; Q-motif (XXXXPTPXQ), ATPase motif (AXXGXGKT, DEAD), the motif involved in ATP binding and cleavage (PTRELA, GG, TPGRL) and the RNA unwinding motifs (SAT) were observed. They were also confirmed in *E. ancora*^11^, *H. magnipapillata*^26^, and *H. echinata*^33^. Thus, *AtVasa* most likely encodes an ATP-dependant RNA helicase. Through partial cloning of *AtLDLR*, on the other hand, typical functional motifs – calcium binding site, putative binding site, epidermal growth factor (EGF)-precursor domains – of LDLR superfamily and cysteine-rich LDLR Domain Class A repeats were observed and are present in chicken^51,52^, teleost^54,55^, insects^40,57–59,67^ and crustaceans^62,63^. Thus, it is likely that *AtLDLR* is a cell-specific lipoprotein receptor. Through partial cloning of *AtVG*, the deduced amino acid sequence contained specific domains – DUF1943 and cleavage sites of subtilisin family endoproteases – that are conserved in the VG of invertebrates^40^ and vertebrates^31^. They were also confirmed in *E. ancora*^45,46^ and *G. fascicularis*^70^. Thus, it is likely that AtVG is one of the major precursors of the egg yolk proteins.

Our histological observations demonstrated that oogenesis is an annual event in *A. tenuis*. Oocytes and spermatocytes disappeared in coral branches after the full moon in June, indicating that the spawning of *A. tenuis* in Okinawa occurs before this moon phase in June. The observation of small oocytes in July indicates that oogenesis begins one month after the mass spawning. We found that vitellogenesis begins in December and actively continues until June. Previous studies of other *Acropora* spp. revealed that the oogenic cycle time varied among different regions. For example, oogenesis in *A. millepora* in the Philippines (~26–28 °C throughout the year)^71^, in broadcasting coral species in the Red Sea (~28 °C in winter; ~34 °C in summer)^72^, and in *Acropora* spp. in the Great Barrier Reef (~29–32 °C in summer)^73,74^ occurred over a period of 8, 6 to 7, and, 9 months, respectively. The shorter oogenic cycle in these regions can be associated with higher seawater temperatures and different climates (e.g., monsoon in the Philippines), which may promote oocyte development in corals. This association may be supported by the fact that warmer water temperatures can increase the rate of development of both gametes^75–77^ and embryos^78,79^ in corals. Furthermore, our results regarding the oogenesis stages correlate with the changes in SST and photoperiod in Okinawa (Fig. 2). As oogenesis requires a significant energy investment, changes in environmental conditions and zooxanthellae photosynthetic activity can influence a colony’s energy reserve and possibly the gametogenic cycle^80–82^. In accordance with this hypothesis, it was demonstrated that the gametogenic cycles of sea anemones and oysters may be accelerated by manipulating seawater temperatures and photoperiod^83–85^. Therefore, it is highly likely that environmental conditions, including day length and water temperature, act as proximate determinants of oogenesis in coral.

*AtVasa* mRNA transcripts were observed in individual cells with small nuclei that were located in the gonadal regions in the retractor muscles of the mesentery along the mesoglea. The transcripts were also expressed in these regions at all stages of oogenesis. Because gametogenesis in corals and cnidarians is known to occur in these regions^9,11^, the cells that are ISH-positive for these transcripts are likely to be germline cells, such as spermatogonia and oogonia. It is possible that these germ cells function as reservoirs for future gametogenic cycles, because they are present throughout the year regardless of the stage of maturation. This statement is supported by a previous study in which early germline cells in *Euphyllia ancora* were not released together with mature gametes during the spawning period, but remained in the mesentery tissues^12^. However, this result differs from the previous study in *Hydra*, in which *Cnvas1* was observed in massive aggregates of germline cells between the ectoderm and mesoglea^26^. A single oocyte was produced within the aggregate and the other germline cells acted as nurse cells which are then consumed by the oocyte. Furthermore, *Cnvas1* and *Cnvas2* of *Hydra*^26^ and *CheVasa* of *Clytia*^32^ were expressed both in oocyte and germline cells, while the expression of *AtVasa* mRNA in oocytes was not observed in the present study. We believe that mRNA of *AtVasa* is not stored as a maternal transcript in the oocyte and might be expressed during different stages of embryogenesis, which could be observed in expression of Vasa (*Nvvas2*) and Nanos (*Nvnos1*) in the starlet sea anemone, *Nematostella vectensis*^9^. On the other hand, maternal expression of Nvvas1 and Nvnos2 are detected in unfertilized and fertilized eggs of *N. vectensis*^9^. In addition, *E.ancora* also demonstrated that *EaVas* and *Eapiwi* gene products (transcripts and proteins) are maternally inherited factors as they were present in unfertilized eggs^11,12^. Nevertheless, it should be taken note that the *AtVasa* mRNA are diluted by other various proteins accumulating within the oocyte cytoplasm, which may cause faint or undetectable ISH signals. During oogenesis, the *A. tenuis* germline cells in the retractor muscles of the mesentery differentiated into oogonia and developed into oocytes freely in the mesoglea. This results differed from the previous study in the starlet sea anemone, in which the oocytes bulge basally into the mesoglea and undergo vitellogenesis while retaining intimate contact with the gastrodermis via trophonema^47,48^.

Localisation of AtVasa immunoreactivity was observed via IHC in the germline cells and in developing oocytes. The observed signals were strong in the germline cells and small immature oocytes, but became weak in the oocyte cytoplasm as the oocytes developed and yolk accumulated. The results of the present study are in agreement with previous reports in *E. ancora*^11^, which found that Vasa protein may play a role in germline cell formation and maintenance, as well as ensuring normal oocyte development. Several reports have demonstrated the involvement of Vasa as a mitotic and meiotic factor in germline cells, as it regulates the localisation of chromosome-associated proteins that mediate chromosome condensation and segregation^27,34,86,87^. Proteins and mRNAs stored in the oocytes may contribute by maximising translation during embryogenesis, as recent reports have shown that Vasa is essential for protein synthesis in early embryos of the sea urchin, *Strongylocentrotus purpuratus*^88^. A decrease in Vasa immunoreactivity in the developing oocyte may be due to an arrest in Vasa protein synthesis and the accumulation of other proteins, leading to the dispersion of Vasa proteins throughout the oocyte cytoplasm^89–91^. It is suggested that other essential proteins (e.g., yolk proteins, ATP synthase, transcription factors, and cyclin B) are synthesised at high levels as the oocyte volume expands and maturation approaches.

ISH and IHC were conducted to determine the localisation of *AtLDLR* transcripts and AtVG immunoreactivity in the coral branches of *A. tenuis*. AtVG immunoreactivity was observed in the cytoplasm of the developing oocytes, putative ovarian sites, and mesentery filaments. The results of the present study agree with those of previous study in *E. ancora*^46^ that yolk protein synthesis in *A. tenuis* is heterosynthetic. It is likely that the immunoreactivity detected in the mesentery (putative ovarian site) cells was synthesised vitellogenin, suggesting that these cells are the major sites of vitellogenin synthesis. Conversely, the immunoreactivity in oocytes was likely yolk, which is a cleavage form of vitellogenin and used as a source of nutrition and energy during embryogenesis after fertilisation^92,93^. It is suggested that the mesoglea of the putative ovarian site is a nutrient storage organ in cnidarians^94^. In contrast, it was suggested that a significant amount of yolk synthesis is autosynthetic within the oocyte due to the presence of synthetic organelles such as rough endoplasmic reticulum and Golgi complexes in developing oocytes during vitellogenesis of the strawberry anemone (*Actinia fragacea*)^95,96^. Aside from vitellogenin, novel egg proteins have been discovered in *G. fascicularis* (GfEP1, GfEP2, GfEP3, and GfEP4)^70^ and *E. ancora* (EaEp and *euphy*)^46,97^. Among the egg proteins identified, GfEP4 in *G. fascicularis* and EaEp and *euphy* in *E. ancora* did not exhibit sequence similarity to VG or proteins identified in other phyla^70,97^, nor did they exhibit immunoreactivity with anti-EaVg/anti-EaEp antibodies^46,97^. It is likely that some proteins with functions other than larval nutrition are present in coral oocytes. The major yolk components of coral oocytes may differ from those of the oocytes of other marine invertebrates and vertebrates. In addition, the gonochoric *G. fascicularis* VG (GfEP-1) were expressed not only in female colonies, but also expressed weakly in male colonies^69^. It was also reported that *G. fascicularis* possess pseudo-oocytes in male colonies^69^, which were not observed in the present study. AtVG immunoreactivity was also not observed in neither testis nor its surrounding tissues (Supplementary Fig. S15). The function of pseudo-eggs is assumed to provide buoyancy for sperm bundles to increase fertilization rate as they are made up of mostly lipids. In case of hermaphroditic corals, the eggs took the role as a buoyant as they are bundled up together with the sperms during maturation before spawning, which explains the absence of pseudo-eggs.

ISH analyses revealed that the *AtLDLR* transcript was present in the mesentery (putative ovarian site) surrounding the oocytes, mesoglea of the mesentery, and mesentery filaments, but not around the membranes of the developing oocytes. This differs from the results of previous studies in other organisms (chicken, rainbow trout, sea bass, and insects), which found the VGR transcript exclusively localised in oocytes within the ovary^52–57^. One possible explanation is that coral oocytes possess a VGR that is different from the VGR of other phyla. This may be supported by the identification of a novel lipoprotein receptor (Lrp13) in white perch (*Morone americana*)^98^ and striped bass (*Morone saxatilis*)^99,100^. Lrp13 specifically binds to only one type of VG out of the three VGs in white perch, and its transcript specifically localised in the oolema and zona radiata of the vitellogenic oocytes. Another possibility is that corals possess multiple LDLRs. Our recent investigations confirmed that *A. tenuis* possess more than one LDLRs (unpublished data), which highlight the possibility of them being VGR. As discussed above, *AtLDLR* belongs to the LDLR superfamily and is responsible for mediating functions in the somatic cells where VG and other egg proteins are synthesised. The LDLR gene superfamily comprises different genes encoding membrane receptors involved in the endocytosis of a variety of ligands. The members of this family that have been characterised in vertebrates include LDLR, LDLR-related proteins, sortilin receptor, and very low-density lipoprotein (VLDL) receptor. The ability of an LDLR to bind to a specific type of ligand (e.g., VG) depends on the ligand binding properties of the receptor^98^. These receptors typically consist of a unique configuration of epidermal growth factor precursors (EGFP), class B YWxD (LDLB), and *O*-linked sugar domains, which define their identities, and class A ligand binding (LDLA) repeats, which determine their particular ligand specificities. A molecular study comparing the presence of a number of ligand binding domain repeats for VGR, LDLR, and VLDL receptor revealed a high degree of similarity (around 85%) among the receptors^101^. Because VGR also belongs to the LDLR superfamily, it shares the same amino acid structure with an eight-repeat ligand binding domain^51,102–104^. In addition to binding to VG, VGRs in the plasma membrane of the oocyte can bind major yolk lipoproteins and VLDL, which are subsequently transported into the cytoplasm of the oocyte^53,101,105^. The mechanisms and components operating in the ovarian tissue of *A. tenuis* may be similar to the LDLR and VLDL receptor systems in somatic tissues of the ones in vertebrates as shown in mouse^102^, chicken^51,101,105^, rabbit^104,106^ and fish^98–100^. We suggest that the *AtLDLR* protein identified in the present study plays an important role in the synthesis of low-density lipoprotein in extraovarian sites and is directly or indirectly involved in oocyte development. It would be interesting to investigate other LDLRs present, as well as the vitellogenesis-related lipoprotein receptors of other vertebrates (such as Lrp13) in corals.

In this study, IHC analyses were performed in oocytes at different stages, revealing that AtVG immunoreactivity detected in oocytes was correlated with the reproductive cycle of *A. tenuis*. Signals were weak at the early stages of oocytes (Stages I and II), but became stronger towards the later stages of gametogenesis (Stages III–V). These characteristics are consistent with the previous reports in *E. ancora*^46^, in which immunoreactivity against VG becomes stronger in oocytes as oogenesis progresses. Additionally, in ISH observations, the expression of *AtLDLR* mRNA was not detected in *A. tenuis* tissues in the immature oocytes stage. However, positive mRNA signals were observed in the somatic tissues of the mesenteries in late Stage II of oogenesis. It is suggested that yolk incorporation starts at Stage III (in November and December) and is activated at Stage IV (March and April) to Stage V (April and May). Histological and IHC analyses of the present study demonstrated that vitellogenesis began after the oocytes entered the mesenterial mesoglea, which is supported by previous reports in *N. vectensis*^47,48^*, G. fascicularis*^69,70^, *E. ancora*^46^ and *Astrangia danae*^107^. It was also reported that vitellogenesis of the sea pen (*Pennatula aculeate*)^108^ and strawberry anemone (*Actinia fragacea*)^95,96^ can be initiated before or after the entry of germ cells into mesoglea. Because the present study revealed that the expression of *AtLDLR* mRNA and immunoreactivity of AtVG changed as oocyte development progressed, *AtLDLR* may be activated as VG is synthesised in the somatic cells, and transported into the oocyte as it develops.

In conclusion, we investigated the expression of the Vasa (*AtVasa*) and LDLR (*AtLDLR*) genes, as well as immunoreactivities against AtVasa and AtVG, in *A. tenuis*. Our results revealed that these proteins are closely related to oogenesis and can be used as markers for oogenesis in corals. Based on the ISH and IHC analyses, it is possible that Vasa plays a role in germ cell determination and development, as well as oogonia-oocyte differentiation. Therefore, the physiological and molecular roles of Vasa last throughout the year. Conversely, the physiological and molecular roles of VG and LDLR are limited to the vitellogenic stages, which last for at least 6 months beginning in December, as these proteins interact with the processes of vitellogenesis, such as lipoprotein transport, vitellogenin synthesis in somatic cells, and incorporation of yolk into oocytes. Because the oogenic cycle of corals was found to correlate with changes in SST and photoperiod, further studies and culture experiments are required to determine the effects of these environmental factors on oocyte development and reproduction-related genes in corals.

## Materials and Methods

### Coral collection

*A. tenuis* samples were collected from reefs (at depth of 2 m during low tide) around Sesoko Island, Okinawa, Japan (26°38′13.964″N, 127°51′56.112″E) by skin diving. Six colonies of *A. tenuis* were tagged, and branches were collected from each colony on a monthly basis for one year (November 2016 to October 2017). Coral branches were collected by removing the branches from the base of the colony using pliers. Healthy coral branches 5–10 cm in length were sampled. Immediately after collection, coral branches were transferred to Sesoko Station, Tropical Biosphere Research Centre of the University of the Ryukyus, Okinawa, Japan. Molecular samples were flash-frozen in liquid nitrogen and stored at −80 °C until further processing. Histological samples were preserved in 4% paraformaldehyde (PFA) (Nacalai Tesque Inc., Kyoto, Japan) for 8 h at 4 °C. The collection of corals was approved by the Okinawa Prefecture government (Nos. 27–76 and 28–84).

### Cloning and characterisation of *AtVasa* and *AtLDLR* cDNAs

*A. tenuis* branch samples were crushed to a fine powder using a liquid nitrogen-cooled mill. Each crushed sample was then mixed with TriPure isolation reagent (Sigma-Aldrich, St. Louis, MO, USA) on ice. Total RNA was extracted according to the manufacturer’s protocol. RNA concentrations were determined using a NanoDrop spectrophotometer (Thermo Fisher Scientific, Waltham, MA, USA). Reverse transcription (RT) was performed to synthesise cDNA from 1000 ng total RNA using a PrimeScript RT reagent Kit with gDNA Eraser (Takara Bio, Kusatsu, Japan), according to the manufacturer’s protocol.

The primer sets for *AtVasa* and *AtLDLR* (Supplementary Table S1) were designed based on the highly conserved regions of the sequences of the *Acropora digitifera* genes in the whole genome database obtained from OIST Marine Genomics Unit (http://marinegenomics.oist.jp)^66^. Partial fragments of *AtVasa* and *AtLDLR* were amplified by long-range polymerase chain reaction (PCR) using the following conditions: LA Taq DNA Polymerase, 35 cycles of denaturation (10 s at 98 °C), annealing (30 s at 60 °C), and extension (2 min at 68 °C). PCR products were subcloned into the pGEM-T Easy vector (Promega, Madison, WI, USA) and transformed into JM109 competent cells (Takara Bio). Plasmid samples were sent to Macrogen Japan (Kyoto, Japan) to determine DNA sequences using a 3730xl DNA analyser (Applied Biosystems, Waltham, MA, USA).

The ORFs of the *AtLDLR* and *AtVasa* nucleotide sequences were identified and then translated into amino acids sequences using a Web-based ORF finder (https://www.ncbi.nlm.nih.gov/orffinder/). The identified ORFs were checked using the Basic Local Alignment Search Tool (BLAST) program (https://blast.ncbi.nlm.nih.gov/Blast.cgi) to confirm the identity of each sequence. The verified amino acid sequences of *AtLDLR* and AtVasa were aligned with related sequences from other closely related cnidarians, other invertebrates, vertebrates, and several further taxa as outgroups using ClustalW^109^. The alignments were then used to construct a phylogenetic tree using the maximum likelihood method with the Jones-Taylor-Thornton (JTT) model^110^ and 1,000 bootstrap replications. The sequence alignment and phylogenetic construction were performed in MEGA7 software^111^.

The gene expression of *AtLDLR* and *AtVasa* was assessed in samples with matured gametes and those with or without immature gametes by amplification using reverse transcriptase polymerase chain reaction (RT-PCR) with the following conditions: 38 cycles of denaturation (45 s at 94 °C), annealing (45 s at 60 °C), and extension (1 min at 72 °C). PCR products were electrophoresed on 2% agarose gel (Wako Pure Chemical, Osaka, Japan) containing ethidium bromide at 110 V for 25 min, and visualised under UV. *A. tenuis β-actin*, a reference gene, was used as a positive control^112^. The PCR product are then subcloned to T-easy vector (Promega) and we sequenced 8 clones to confirm that PCR successfully amplified the targeted cDNAs.

### Histological analyses

After preservation, the coral branches were decalcified with Morse’s solution for 3–5 days. Following dehydration in a graded RNAse-free ethanol series and permutations with xylene, portions of the coral tissue were embedded in histoparaffin (Surgipath Paraplast, Leica Biosystems, Nussloch, Germany; melting point 56 °C), serially sectioned at 4 μm, and then stained with haematoxylin and eosin Y (H & E staining) for microscopic observations. The development of gametes was scored and classified according to cell size and histological characteristics with brief modifications from the previously reported method^11^ (Supplementary Table S2). For measurements of oocyte diameter, oocytes (n = 60–70 oocytes from each colony) with visible nuclei were randomly chosen. Their maximum diameter length was measured, and a second measurement was taken of the axis passing perpendicularly through the first axis. All oocyte measurements were obtained using ImageJ64 software (National Institutes of Health, Bethesda, MD, USA). The oocyte diameters presented are expressed as the geometric mean of two measurements, which was calculated using the formula below:

$${\rm{Geometric}}\,{\rm{mean}}=\sqrt{{\rm{Maximum}}\,{\rm{length}}\times {\rm{Width}}\,{\rm{perpendicular}}\,{\rm{to}}\,{\rm{\max }}\,.\,{\rm{length}}}$$

The stages of oogenesis were classified and characterised as follows:

#### Post spawning stage

After mass spawning, the coral enters this stage in June. No oocytes are visible in the mesentery. Traces of the spawned oocytes are seen as distinct gaps or spaces in the mesentery, and appear stretched and distorted in shape (Supplementary Fig. S14).

*Stage I*: Small oocyte ranging from 35 to 75 µm in diameter can be observed alongside the mesoglea of the mesentery in July. Oocytes are still not visible, even under a dissection microscope. The oocyte cytoplasm (purplish in colour) and nucleus (pink in colour) can be observed (Fig. 1a).

*Stage II*: Oocyte ranging from 76 to 175 µm in diameter can be observed within the mesoglea. As the oocyte diameter increases, the cytoplasm and nucleus of the oocyte increase in size. The oocytes can be seen as pairs or triplets per mesentery. As lipid vesicles accumulate, fine or coarse granular matter appears in the cytoplasm of each oocyte. The ooplasm still stains purple and the nucleus stains pink (Fig. 1b).

*Stage III*: Oocytes migrated into the mesoglea of the mesentery. Oocytes ranging from 176 to 285 µm in diameter can be observed. Oocytes are visible under a dissection microscope. The yolk can be observed in the cytoplasm as granules with a slight purplish-red colour. Due to the accumulation of yolk in the cytoplasm, the size of the oocyte increases significantly (*P* < 0.05). Yolk polarity in the cytoplasm and peripheral migration of the nucleolus in the nucleus are observed (Fig. 1c).

*Stage IV–V*: The oocyte size increases significantly to 286 to 385 µm in diameter at stage IV and reaches the full size of >386 µm at stage V. The yolk becomes almost homogenous in the cytoplasm, which has a pinkish-red to red colour with a little purplish hue. The nucleus appears darker red in colour in the centre of the cytoplasm (Fig. 1d,e).

*Stage VI*: Oocytes are differentiated into eggs, which are released as bundles during mass spawning. The egg (red in colour) reaches 600 µm in diameter. The yolk and lipid droplets are visible in histological observation (Fig. 1f).

### ISH

In this study, ISH was used localise the expression of *AtVasa* and *AtLDLR* mRNA in *A. tenuis* tissues. Digoxigenin (DIG)-labelled sense and antisense probes were synthesised using partial *AtVasa* and *AtLDLR* cDNA fragments (350–500 bp) via *in vitro* transcription (Supplementary Table S1,S3). Transcription was performed using 100 ng of linearised plasmid in the presence of purified template DNA and RNA Polymerase SP6 or T7. The probes were then purified with the Agencourt RNAClean XP Kit (Agencourt) and stored at −30 °C until further use.

Additional two pairs of PCR-based method RNA probes^113^ of each genes were synthesised and tested to confirm the specificity of the RNA probes. Primary amplified products of PCR containing the specific DNA sequences were synthesised from cDNA. For the secondary PCR amplification, T7 promoter sequences were added before the target DNA sequences. The purity and size of the PCR products were verified by gel electrophoresis. The DIG-labeled probes were synthesised by *in vitro* transcription in the presence of purified template DNA (with T7 promoter sequence) and RNA polymerase T7. The probes were then purified with the Agencourt RNAClean XP Kit (Agencourt Bioscience Corporation, Beverly, MA, USA) and stored at −30 °C until further use.

Coral tissues embedded in paraffin were cut into 4-μm serial sections for anti-sense, sense, and H & E stains. The sections were deparaffinised with xylene, hydrated, and processed for ISH with DIG-labelled RNA probes. The hydrated sections were subjected to digestion with proteinase K (10 μg/mL) at 37 °C for 15 min, fixation with 4% PFA, and then pre-hybridised with 2× SSC/50% formamide. Next, the sections were hybridised with a hybridisation mixture of 500 ng/mL DIG-labelled RNA probes, 10% dextran sulphate, 2× SSC, 0.02% SDS, and 50% formamide. After hybridisation at 60 °C for 16 h, the sections were washed as follows: once in 2× SSC at room temperature for 5 min, 3 times in 1× SSC/50% formamide at 60 °C for 30 min, 3 times in 1× SSC at 60 °C for 30 min, and 2 times in TBS for 5 min.

The sections were immersed in blocking solution containing 10% bovine serum albumin, which was mixed with maleic acid for 1 h at room temperature. The DIG-labelled RNA probe was detected using anti-DIG-AP Roche, Basel, Switzerland antibody, which was diluted 1:1000 with the same blocking solution for 1 h at room temperature or overnight at 4 °C. The immunoreactive signals were visualised using the NBT/BCIP liquid substrate system (Sigma-Aldrich). Sections were observed and photographed under a microscope.

### IHC

IHC was performed to localise Vasa and VG proteins in *A. tenuis* tissues. Polyclonal antibodies against Vasa and VG were gifted from National Taiwan Ocean University (Keelung, Taiwan) and were generated against the polypeptides C + AVGTSYGPAGGRFASRD (anti-EaVas) and CGDADGEQWNEYKDPQGR (anti-EaVg) of *E. ancora*. The polypeptides were conjugated to ovalbumin and immunised to rabbit (anti-EaVas) and guinea pig (anti-EaVg). The antiserum was purified using an affinity column containing 3 mg of the respective peptide antigen (Yao-Hong Biotechnology Inc., Taipei, Taiwan)^11,46^. The avidin-biotinylated-peroxidase complex (ABC) kit (Vector Laboratories, Burlingame, CA, USA) was used to detect immunoreactivity against antibodies to Vasa and VG. The antibody specificity was confirmed by Western blotting (Supplementary Fig. S16).

Coral tissues embedded in paraffin were cut into 4-μm sections. The sections were deparaffinised with xylene and hydrated in an ethanol hydration series, followed by incubation in HistoVT One antigen retrieval solution (Nacalai Tesque, Kyoto, Japan) for antigen retrieval. The sections were then incubated for 10 min in 3% H_2_O_2_ and blocked with goat serum for 1 h. After that, the sections were immersed in purified primary antibodies (1:4000 in PBS, with goat serum) and incubated for 16 h (or overnight) at 4 °C. After washing with PBS, the sections were incubated with a secondary biotinylated goat anti-rabbit/anti-guinea pig IgG antibody (Vector Laboratories; diluted 1:2000) for 30 min. Then, the sections were incubated in ABC solution and visualised using 3–3′-diaminobenzidine (DAB; Sigma-Aldrich). For control samples, no primary antibodies were added (only serum and PBS were used). The sections were observed and photographed under a microscope.

### Statistical analysis

Data are shown as the mean ± standard error of the mean (SEM). Statistical significance was analysed using RStudio software (Integrated Development for R, RStudio, Inc., Boston, MA, USA; http://www.rstudio.com). One-way analysis of variance (ANOVA) and Kruskal-Wallis non-parametric analyses were applied according to Bartlett’s homogeneity and the Shapiro-Wilk normality test. Multiple pairwise analyses using Tukey’s honestly significant difference (HSD) test were applied to compare means among analysed groups. The statistical significance level was set to *P* < 0.05.

## Supplementary information

Supplementary Figures.

## Data Availability

The datasets generated during and/or analysed during the present study are included in this published article (and its Supplementary Information files). Further enquiry on other datasets are available from the corresponding author on reasonable request.
