# Supplementary material for: Molecular approaches underlying the oogenic cycle of the scleractinian coral, Acropora tenuis
Source: Sci Rep. 2020 Jun 18;10:9914. doi: 10.1038/s41598-020-66020-x (PMC7303178; doi:10.1038/s41598-020-66020-x)
Supplement: Supplementary file 1 — Supplementary Figures. [file 41598_2020_66020_MOESM1_ESM.docx]

**Molecular approaches underlying the oogenic cycle of the scleractinian coral, *Acropora tenuis***

**Ee Suan Tan^1^, Ryotaro Izumi^1^, Yuki Takeuchi^2,3^, Naoko Isomura^4^, & Akihiro Takemura^2,^***

^1^ Graduate School of Engineering and Science, University of the Ryukyus, Nishihara, 903-0213, Japan

^2^ Department of Chemistry, Biology and Marine Science, Faculty of Science, University of the Ryukyus, Nishihara, 903-0213, Japan

^3^ Developmental Neurobiology Unit, Okinawa Institute of Science and Technology Graduate University (OIST), Onna, 904-0412, Japan

^4^ Department of Bioresources Engineering, National Institute of Technology, Okinawa College, Nago, 905-2192, Japan

*Correspondence should be addressed to A.T. (takemura@sci.u-ryukyu.ac.jp)

| Primer | Sequence | Reference |
| --- | --- | --- |
|  |  |  |
| *Partial cloning primers* |  |  |
| *AtVasa - Forward* | 5'-CGGAGCTAATGGTTCTCCAG-3' |  |
| *AtVasa - Reverse* | 5'-GGTTCAAATCCCATGTCCAG-3' |  |
| *AtLDLR - Forward* | 5'-TCGGAGGCATTCTGTAATCC-3' |  |
| *AtLDLR - Reverse* | 5'-CATCCACGACATCAGTCCAG-3' |  |
| *AtVG - Forward* | 5'-CCACTGCGAACTTTGGTGTG-3' |  |
| *AtVG - Reverse* | 5'-CTTTCCTCCTGGCATTTGCG-3' |  |
| *β-actin* *- Forward* | 5'-GCAAAAGGAAATTGCTGCTC-3' | No. 112 |
| *β-actin* *- Reverse* | 5'-CACATCTGTTGGAAGGTGGA-3' | No. 112 |
|  |  |  |
| *In-fusion cloning primers* |  |  |
| *AtVasa - Forward* | 5'-CTATAGGGCGAATTGCGGAACTGGATCCAAATCGC-3' |  |
| *AtVasa - Reverse* | 5'-AGAATACTCAAGCTAACAGGCGAGTTCTCGAGTTG-3' |  |
| *AtLDLR - Forward* | 5'-CTATAGGGCGAATTGCTCGCCCTCTTTGCAAACAC-3' |  |
| *AtLDLR - Reverse* | 5'-AGAATACTCAAGCTACACTTGTGTGAACAGTGGCC-3' |  |
|  |  |  |
| *ISH Probe template primers* |  |  |
| *AtVasa - Forward* | 5'-CGGAACTGGATCCAAATCGC-3' |  |
| *AtVasa - Reverse* | 5'-ACAGGCGAGTTCTCGAGTTG-3' |  |
| *AtLDLR - Forward* | 5'-CTCGCCCTCTTTGCAAACAC-3' |  |
| *AtLDLR - Reverse* | 5'-CACTTGTGTGAACAGTGGCC-3' |  |
|  |  |  |

**Table S1**. Primers used in the present study.

**Table S2**. Criteria for classification of stages in oocyte development, as observed in histological sections.

| Stage | Oocyte |
| --- | --- |
| PS | No visible oocytes in mesentery. |
| I | Oocytes of 35-75 µm in diameters. |
| II | Oocytes of 76-175 µm in diameters. |
| III | Oocytes of 176-285 µm in diameters. |
| IV | Oocytes of 286-385 µm in diameters. |
| V | Oocytes of >386 µm in diameters. |

*PS: post spawning

**Table S3**. Primers used for PCR-based RNA probe.

| Primer | Sequence |
| --- | --- |
|  |  |
| *ISH probe primers (PCR-based)* |  |
| *AtVasa anti-sense Forward 1* | 5'-ACAGCGTAATACGACTCACTATAGGGATCCTGTCTGAGCACATGCC-3' |
| *AtVasa anti-sense Reverse 1* | 5'-AGTGGATTTGGTGGAGGTGG-3' |
| *AtVasa sense Forward 1* | 5'-ACAGCGTAATACGACTCACTATAGGGAGTGGATTTGGTGGAGGTGG-3' |
| *AtVasa sense Reverse 1* | 5'-ATCCTGTCTGAGCACATGCC-3' |
| *AtVasa anti-sense Forward 2* | 5'-ACAGCGTAATACGACTCACTATAGGGACAGGCGAGTTCTCGAGTTG-3' |
| *AtVasa anti-sense Reverse 2* | 5'-GAGGGACATTTTGCTCGGGAG-3' |
| *AtVasa sense Forward 2* | 5'-ACAGCGTAATACGACTCACTATAGGGAGGGACATTTTGCTCGGGAG-3' |
| *AtVasa sense Reverse 2* | 5'-ACAGGCGAGTTCTCGAGTTG-3' |
|  |  |
| *AtLDLR anti-sense Forward 1* | 5'-ACAGCGTAATACGACTCACTATAGGGCTCGCCCTCTTTGCAAACAC-3' |
| *AtLDLR anti-sense Reverse 1* | 5'-CAGCGATGAACAAGGCTGTG-3' |
| *AtLDLR sense Forward 1* | 5'-ACAGCGTAATACGACTCACTATAGGGCAGCGATGAACAAGGCTGTG-3' |
| *AtLDLR sense Reverse 1* | 5'-CTCGCCCTCTTTGCAAACAC-3' |
| *AtLDLR anti-sense Forward 2* | 5'-ACAGCGTAATACGACTCACTATAGGGATCAGTCCAGAACACCTGCC-3' |
| *AtLDLR anti-sense Reverse 2* | 5'-GTGTTTGCAAAGAGGGCGAG-3' |
| *AtLDLR sense Forward 2* | 5'-ACAGCGTAATACGACTCACTATAGGGGTGTTTGCAAAGAGGGCGAG-3' |
| *AtLDLR sense Reverse 2* | 5'-ATCAGTCCAGAACACCTGCC-3' |
|  |  |

A. tenuis IERGINFDKYDEIPVEVTGSGKESIIPIQSFSQAQLYDTFQQNVKKS**NYTKPTPVQ**KYAI 272

**Q-motif**

A. digitifera IERGINFDKYDEIPVEVTGRGKESIIPIQSFSQAQLYDTFQQNVKKS**NYTKPTPVQ**KYAI 64

S. pistillata IERGINFDKYDEIPVEVTGKGKEAIIPIQTFQEANLRNLLLENVQKA**KYTKPTPVQ**KYAI 302

O. faveolata IERGINFDKYDEIPVEVTGKGKESITPIQKFEQAGLREKLQENVAKI**GYTKPTPVQ**KYAI 344

E. ancora IERGINFDKYDEIPVEVTGRGKEAIIPIQGFHQAQLYETFQGNVKKA**GYTKPTPVQ**KYAI 254

******************* ***:* *** * :* * : : ** * ************

**ATPase motif**

**ATP binding and cleavage**

A. tenuis PAILGGRDVMAC**AQTGSGKT**AAFLLPVMTGMLQKGLTSSSMMGGPQCPQALIIS**PTRELA** 332

A. digitifera PAILGGRDVMAC**AQTGSGKT**AAFLLPVMTGMLQKGLTSSSMMGGPQCPQALIIS**PTRELA** 124

S. pistillata PTIIAGRDVMAC**AQTGSGKT**AAFLLPAMSGILNSGLTSSELS-GIQTPQGLCIS**PTRELA** 361

O. faveolata PCILAGRDVMAC**AQTGSGKT**AAFLLPVMDGMLNSGLTSSELS-ATQTPQALCIS**PTRELT** 403

E. ancora PAILAGRDVMAC**AQTGSGKT**AAFLLPVMTGMLQKGLTSSAMTAGAHSPQALIIS**PTRELA** 314

* *:.*********************.* *:*:.***** : . : **.* *******:

**ATP binding and cleavage**

A. tenuis CQIYNEARKFSYETIVKTVVVY**GG**VSVPHQLRKI-EMGCNLLVA**TPGRL**KDFIERGKISL 391

A. digitifera CQIYNEARKFSYETIVKTVVVY**GG**VSVPHQLRKI-EMGCNLLVA**TPGRL**KDFIERGKISL 183

S. pistillata SQIYNEARKFSKGTMLKPVCIY**GG**VSVSHQLRQV-QRGCNFLVA**TPGRL**KHFVEGGQVSL 420

O. faveolata TQIYNEARKFSHQTMLRPAVAY**GG**VSVAHQLRHI-ERGCNLLVA**TPGRL**LDFVERGKVSL 462

E. ancora LQIYNEARKFSHSTMLVPAVAY**GG**VSVQHQLRQLQNKGCNLLVA**TPGRL**ADFVEKDRISL 374

********** *:: . ****** ****:: : ***:******** .*:* .::**

**RNA unwinding motif**

**ATPase motif**

A. tenuis EKIQYLIL**DEAD**RMLDMGFE---------------------------------------- 411

A. digitifera EKIQYLIL**DEAD**RMLDMGFEPVIRQVVEKLGMPGKCERQTLMF**SAT**FPEEIQRLAGDFLN 243

S. pistillata EKIQYLIL**DEAD**RMLDMGFEGDIRKIVETLGMPEKTERQTLMF**SAT**FPEEIQRLAGDFLN 480

O. faveolata QCIQYLIL**DEAD**RMLDMGFEPSIRKLVETMGMPEKADRQTLMF**SAT**FPEEIQRLAGDFLN 522

E. ancora KAVQYLIL**DEAD**RMLDMGFEPKIRSIVENMGMPAKSERQTLMF**SAT**FPEEIQRLAGDFLN 434

: :*****************

**Fig. S1**. Amino acid alignment (Vasa) of *A. tenuis*, other scleractinians including *Acropora digitifera* (AN4224), *Stylophora pistillata* (XP_022783312.1), *Orbicella faveolata* (XP_020624779.1) and *Euphyllia ancora* (AFP52950.1)*.* Asterisks (*) indicate a perfect match among all aligned sequences, whereas dots represent conservative replacements: (.) conserved; (:) highly conserved. Domain and conserve motif region were indicated on the top of sequence and highlighted in orange.


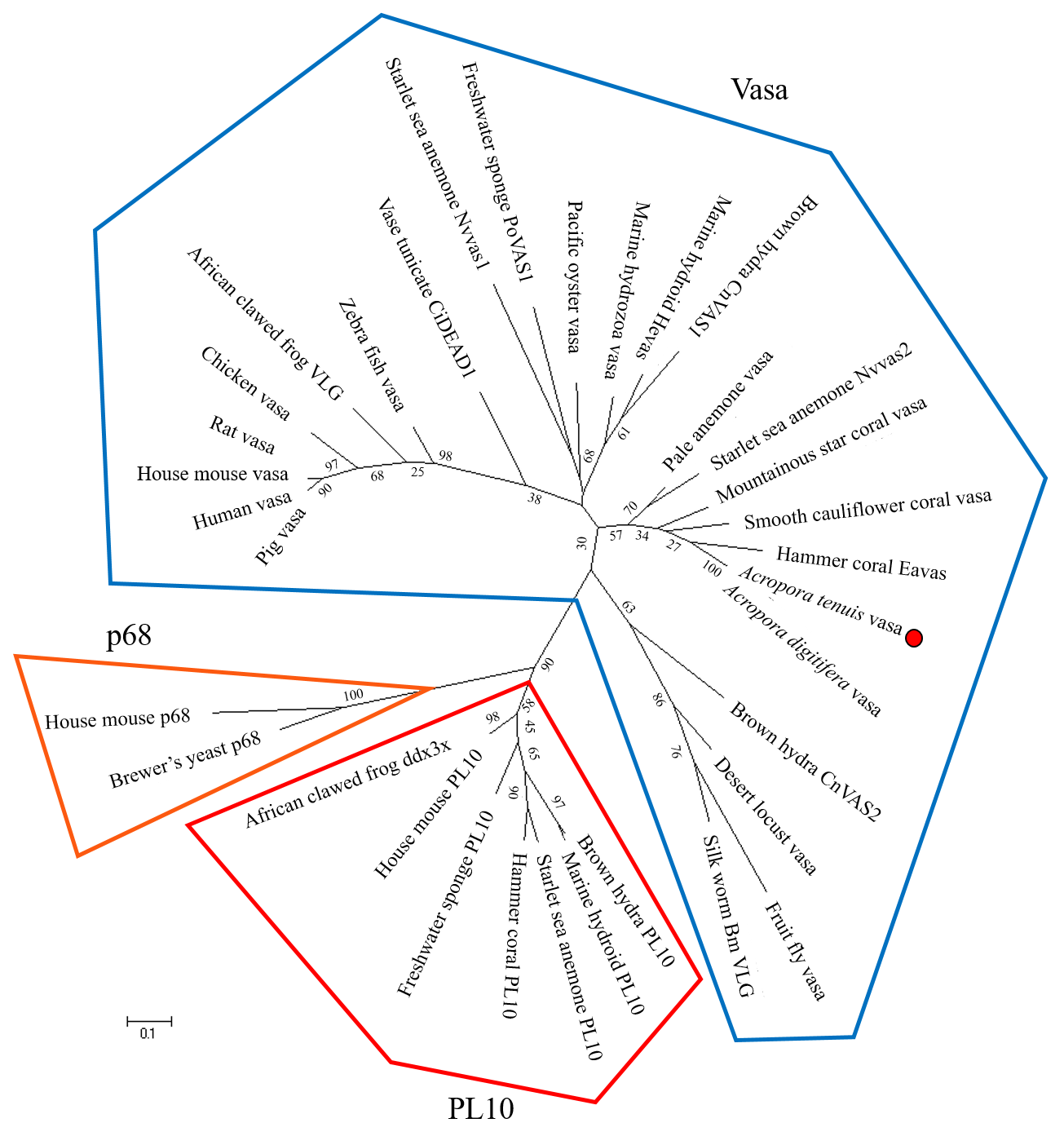


**Fig. S2.** Phylogenetic analysis comparing the amino acid sequences of Vasa, PL10, and p68 of various taxa. The consensus tree with 1000 bootstrap replications was obtained using the Maximum likelihood analysis with Jones-Taylor-Thornton (JTT) model. The sequences were aligned by multiple sequence alignment using MEGA7.0. Bootstrap values were expressed as percentages; the branches shown correspond to values of 20% and higher. Red circle express AtVasa. The scale bar is calibrated in substitutions per site. The names and corresponding GenBank accession numbers of the proteins analysed are as follows: Smooth cauliflower coral vasa (*Stylophora pistillata*) XP_022783312.1, Mountainous star coral vasa (*Orbicella faveolata*) XP_020624779.1, Hammer coral vasa (*Euphyllia ancora*) AFP52950.1, *Acropora tenuis* vasa, *Acropora digitifera* vasa AN4224, Starlet sea anemone vasa (*Nematostella vectensis*) AAW29073.1 & AAW29074.1, Pale anemone vasa (*Exaiptasia diaphana*) XP_020898583*,* Freshwater sponge vasa (Ephydatia fluviatilis) BAB13310.1, Pacific oyster vasa (*Crassostrea gigas*) NP_001292258.1, Marine hydroid vasa (*Hydractinia echinata*) BAB13686.1, Brown hydra vasa (*Hydra vulgaris*) BAB13307.1 & BAB13308.1, Marine Hydrozoa vasa (*Tima formosa*) BAB13687.1, Vase tunicate vasa (*Ciona intestinalis*) BAA36710.1, House mouse vasa (*Mus musculus*) BAA03584.1, Rat vasa (*Rattus sp.*) AAB33364.1, Human vasa (*Homo sapiens*) NP_077726, Pig vasa (*Sus scrofa*) Q6GWX0.1, Chicken vasa (*Gallus gallus*) BAB12337.1, Zebra fish vasa (*Danio rerio*) AAI29276.1, African clawed frog vasa (*Xenopus laevis*) NP_001081728.1, Desert locust vasa (*Schistocerca gregaria*) AAO15914.1, Silk worm vasa (*Bombys mori*) BAA19572.1, Fruit fly vasa (*Drosophila melanogaster*) BAA03584.1, Hammer coral PL10 (*Euphyllia ancora*) JQ968406, Starlet sea anemone PL10 (*Nematostella vectensis*) AAW29072, Marine Hydroid PL10 (*Hydractinia echinata*) BAB13679, Brown hydra PL10 (*Hydra vulgaris*) BAB13306, Freshwater sponge PL10 (*Ephydatia fluviatilis*) BAB13309, Zebra fish PL10 (*Danio rerio*) NP_571016, African clawed frog ddx3x (*Xenopus laevis*) NP_001080283, House mouse PL10 (*Mus musculus*) AAA39942, House mouse p68 (*Mus musculus*) CAA46581, and Brewer’s yeast p68 (*Saccharomyces cerevisiae*) CAA36874.

A.tenuis ---------EAFCNPATKFQ**C**KTTER**C**IPQRWL**C**DSDD**DC**GD--NSDEPPD**C**TQRT**C**SDR 49

**Calcium binding site**

**LDLPR Domain Class A**

A.digitifera **NHSDE**RGCSEAFCNPDTKFQ**C**KTTER**C**IPQRWL**C**DSDD**DC**GD--NSDEPPD**C**TQRT**C**SDG 113

S.pistillata **DNSDE**KNCPVRHCDLSTHFE**C**KTVDR**C**VPQRWV**C**DFAN**DC**GD--NSDEPAN**C**AERT**C**SQN 115

N.vectensis **DGSDE**QGS--PKTCLQDQFT**C**RN-GK**C**IQATWK**C**DGED**DC**RDGYRSDE-SN**C**GNVT**C**GAD 84

H.sapiens **DGSDE**KNC-VKKTCAESDFV**C**NN-GQ**C**VPSRWK**C**DGDP**DC**EDG-SDESPEQ**C**HMRT**C**RIH 116

.* *.. :*: * ** ** * .:. :* **

**Putative binding site**

A.tenuis EHA**C**GNG--H**C**IPLRWV**C**DADKD**C**S**DNSDE**QG**C**GNKT**C**KTSEFT**C**SNGK**CI**SQAWV**C**DHD 107

A.digitifera EHA**C**GNG--H**C**IPFRWV**C**DGDKD**C**S**DNSDE**QG**C**GNKT**C**KGSEFT**C**SNGK**CI**SQAWV**C**DHD 171

S.pistillata EYA**C**GNG--H**C**IPMRWR**C**DHDKD**C**E**DGSDE**RD**C**GNKT**C**DGNQFT**C**GNGK**CI**SRSWV**C**DRD 173

N.vectensis EFM**C**SNR--K**C**ISRSWT**C**DNQDD**C**G**DNSDE**DRNVQRT**C**ASNQFT**C**SNGD**CI**SNSWT**C**DGD 142

H.sapiens EIS**C**GAHSTQ**C**IPVSWR**C**DGEND**C**D**SGEDE**EN**C**GNIT**C**SPDEFT**C**SSGR**CI**SRNFV**C**NGQ 176

* *. :**. * ** :.** ...** : ** .:***..* ***. :.*: :

A.tenuis N**DC**D**DGSDE**LH--**C**PNAT**C**KPTQFT**C**VNS-GR**C**IKQEWK**C**DGDND**C**Q**DNFDE**--LG**C**PTL 162

A.digitifera N**DC**D**DGSDE**WH--**C**PNVT**C**KPTQFT**C**VNN-GR**C**IKQEWK**C**DGDND**C**Q**DNYDE**--LG**C**PTR 226

S.pistillata D**DC**T**DYSDE**RN--**C**PNKT**C**KPTEFT**C**NN--GK**C**IKASYK**C**DGDND**C**Q**DGSDE**--LG**C**PTT 227

N.vectensis N**DC**N**DGSDE**KESL**C**ASKS**C**KITEFT**C**RTSRRK**C**IPSQWK**C**DGDND**C**P**DSSDE**--SG**C**PTA 200

H.sapiens D**DC**S**DGSDE**LD--**C**APPT**C**GAHEFQ**C**STS--S**C**IPISWV**C**DDDAD**C**S**DQSDE**SLEQ**C**GRQ 232

:** * *** . *. :* :* * . ** .: **.* ** * ** *

A.tenuis SP---R**C**KESQFQ**C**KLKSQ**C**IDSSWV**C**DGEED**C**Q**DRTDE**KE**C**DQRT**C**PPNHFT**C**ANGR**C**I 219

A.digitifera SP---K**C**KESYFQ**C**KLNFQ**C**IDSSWV**C**DGEPD**C**Q**DETDE**KE**C**DKIT**C**PPNHFT**C**ANGR**C**I 283

S.pistillata GP---T**C**KYSEFQ**C**KS-HE**C**IHESWI**C**DGEND**C**S**DGSDE**KG**C**NVTS**C**GPSEFS**C**TNGR**C**I 283

N.vectensis SVSPRR**C**SVGMFK**C**RN-GE**C**VLGHWR**C**DGEKD**C**S**DGSDE**KG**C**RKSN**C**ASSEFT**C**ANGQ**C**I 259

H.sapiens PVIHTK**C**PASEIQ**C**GS-GE**C**IHKKWR**C**DGDPD**C**K**DGSDE**VN**C**PSRT**C**RPDQFE**C**EDGS**C**I 291

* . ::* :*: * ***: **.* :** * .* ...* * :* **

A.tenuis AQQKW**C**DGIDD**C**L**DDSDE**QG**C**PLPTPSV**C**KEGEFQ**C**GDSNQ**C**IKDQKV**C**DNHKD**C**HN-GK 278

A.digitifera AEQKW**C**DGIDD**C**L**DDSDE**QK**C**PLPTPSV**C**KEGEFQ**C**GDSNQ**C**VKNQDV**C**NNHKD**C**HN-GE 342

S.pistillata TIHSQ**C**DGRND**C**L**DNSDE**QD**C**APVKPKP**C**KKDEFQ**C**GNTEQ**C**IPYSKV**C**DGNAD**C**KNRLD 343

N.vectensis PSSQR**C**DGTSN**C**R**DSSDE**KA**C**VTPPP--**C**MPGEFK**C**QSTGR**C**IPESKV**C**DGTRD**C**QD-GE 316

H.sapiens HGSRQ**C**NGIRD**C**V**DGSDE**VN**C**KNVNQ-CLGPGKFK**C**R-SGE**C**IDISKV**C**NQEQD**C**RDWSD 349

*:* :* *.*** * .:*:* : .*: ..**: **:: .

**Epidermal growth factor**

A.tenuis DEPES**C**GKNE**C**LEHNGH**C**QQL**C**NDTKDSFF**C**S**C**RPGYKVHPKEHNI**C**IDVDE**C**DTLGT**C**S 338

A.digitifera DEPES**C**GKNE**C**LEHNGH**C**QQL**C**NDTKDSYF**C**S**C**RPGYMVHPKEHNI**C**IDVDE**C**DTLGT**C**S 402

S.pistillata DEPLS**C**SINE**C**NYHNGH**C**QQI**C**TDTKTSYI**C**S**C**RVGYKVDKDDRRV**C**RDVDE**C**EIPGS**C**S 403

N.vectensis DEPLR**C**NIDE**C**KDHNGH**C**SQK**C**NDLTLGYN**C**S**C**FSGYKLQG--ARL**C**VDIDE**C**AEYGT**C**S 374

H.sapiens EPLKE**C**HINE**C**LVNNGG**C**SHI**C**KDLVIGYE**C**D**C**AAGFELID--RKT**C**GDIDE**C**QNPGI**C**S 407

: * :** :** *.: *.* .: *.* *: : . * *:*** * **

A.tenuis QV**C**YNTKGSFK**C**L**C**CEGYLMEPDQRT**C**KAKGGEGFLVFANRHDIRQLAFDSSDYTEVIPD 398

A.digitifera QV**C**YNTKGSFK**C**L**C**CEGYLMEPDQRT**C**KAEGGEGFLVFANRHDIRQLAFDSSDYTEVIPD 462

S.pistillata QA**C**YNQKGSFK**C**I**C**RNGYVMEPDRQS**C**KAIGEPGFLIFANRQDIRHLSFDSSDYREMVPE 463

N.vectensis QV**C**ENRKGSFK**C**S**C**LPGYRIDGDGRT**C**RANGTLPSLVYSSQFSIRNVTVSGAISQAIVSG 434

H.sapiens QI**C**INLKGGYK**C**E**C**SRGYQMDLATGV**C**KAVGKEPSLIFTNRRDIRKIGLERKEYIQLVEQ 467

* * * **.:** * ** :: *:* * *:::.: .**:: .. ::

A.tenuis LRGAIALDFDFESGQVFWTDVVD------------------------------------- 421

A.digitifera LKGAIALDFDYESRQVFWTDVVDENIKSAKMEPNPTVQPLANVSLDTPDGIAVDWINKKL 522

S.pistillata QRGAIALDYDFESGYIFWTDVIDENIRRTKMHDTSPVEDLVKINLDTPDGIAVDWINRKL 523

N.vectensis RKGVVGLDYDYKSNLIFWTDAKAEKINRARLDGSGSVEEIVG-DVKVPDDVTVDWSGRKI 493

H.sapiens LRNTVALDADIAAQKLFWADLSQKAIFSASIDDKVGRHVKMIDNVYNPAAIAVDWVYKTI 527

:..:.** * : :**:*

**Fig. S3.** Amino acid alignment (LDLR) of *A. tenuis*, other cnidarians including *Acropora digitifera* (AN6885), *Stylophora pistillata* (PFX18088.1), *Nematostella vectensis* (XP_001635789.1) and human, *Homo sapiens* (AAA61344.1)*.* Asterisks (*) indicate a perfect match among all aligned sequences, whereas dots represent conservative replacements: (.) conserved; (:) highly conserved. Domain region and motifs were indicated on the top of sequence. Calcium binding site; enclosed dash box, putative binding; dotted lines, epidermal growth factor; solid line, D-X-S-D-E motif; green highlight, cysteine chain; yellow highlight, F-W-T-D motif; turquoise highlight.


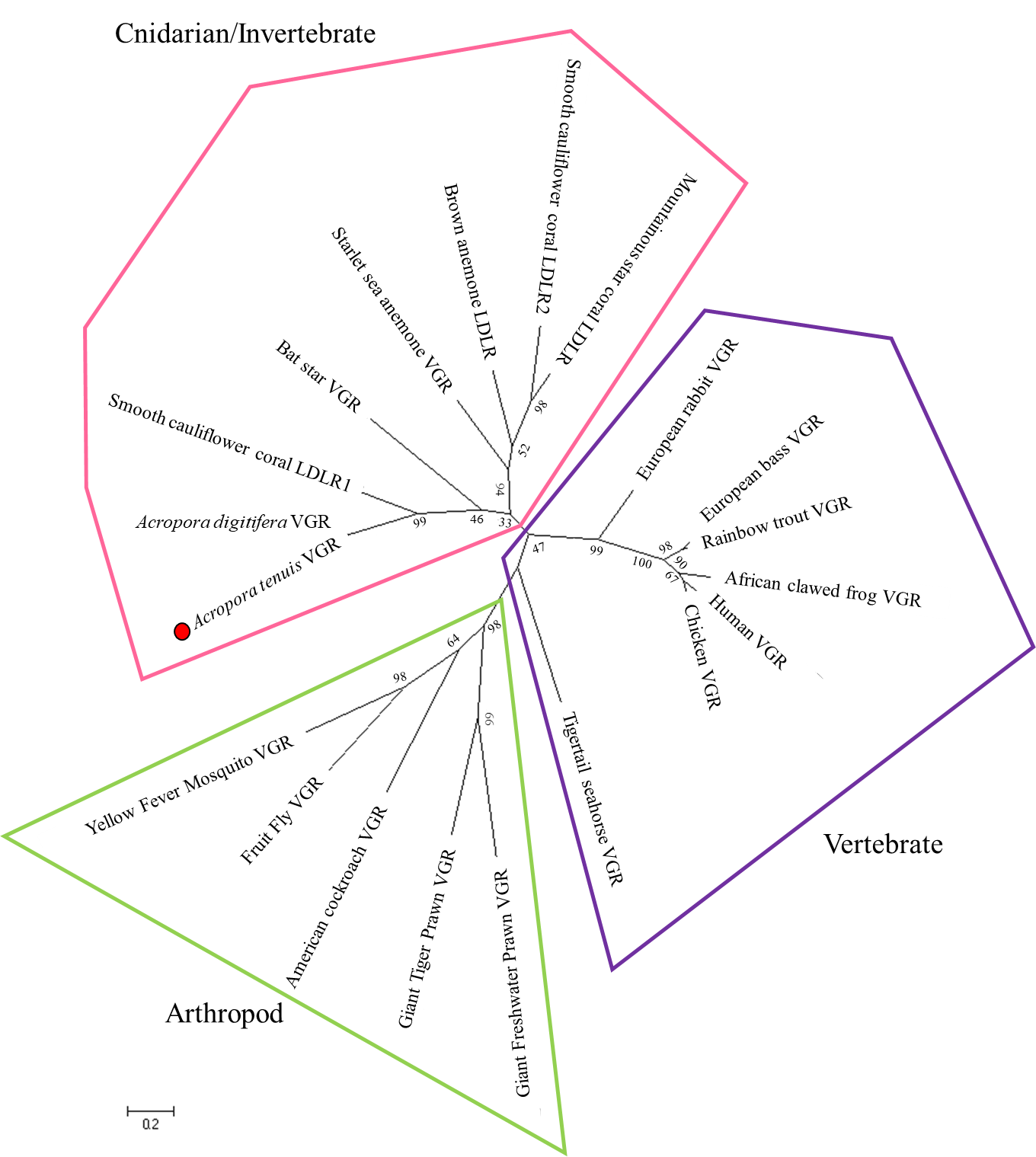


**Fig. S4**. Phylogenetic analysis comparing the amino acid sequences of VGR/LDLR of various taxa. The consensus tree with 1000 bootstrap replications was obtained using the Maximum likelihood analysis with Jones-Taylor-Thornton (JTT) model. The sequences were aligned by multiple sequence alignment using MEGA7.0. Bootstrap values were expressed as percentages. Red circle express *AtLDLR*. The scale bar is calibrated in substitutions per site. The names and corresponding GenBank accession numbers of the proteins analysed are as follows: European bass (*Dicentrachus labrax*) CBX54721.1, Rainbow trout (*Oncorhynchus mykiss*) CAA05873.1, African clawed frog (*Xenopus laevis*) BAA22145.1, Human (*Homo sapiens*), Chicken (*Gallus gallus*) NP_990560.1, European rabbit (*Oryctolagus cuniculus*) NP_001265794.1, Tigertail seahorse (*Hippocampus comes*) XP_019731953.1, *Acropora tenuis*, *Acropora digitifera* (AN6885), Smooth cauliflower coral (*Stylophora pistillata LDLPR1*) PFX18088.1 & PFX20360.1, Starlet sea anemone (*Nematostella vectensis*) XP_001635789.1, Brown anemone (*Exaiptasia diaphana*) XP_020901735.1, (Mountaineous star coral (*Orbicella faveolata*) XP_020606236.1, Bat star (*Patiria miniata*) AMR68937.1, Giant freshwater prawn (*Macrobrachium rosenbergii*) ADK55596.1, Giant tiger prawn (*Penaeus monodon*) ABW79798.1, American cockroach (*Periplaneta americana*) BAC02725.2, Fruit fly (*Drosophila melanogaster*) AAB60217.1, Yellow fever mosquito (*Aedes aegypti*) AAK15810.1.

G.fascicularis NQLEYPGEGITISKGGVEIKQTLEKAFMDANAQNVLPTLAGIPLNIQFRSAASVRITAGA 893

E.ancora NQLEYPGEGITISKEGVEIKQTLEKAFMDANAQRVLPTLAGILLNLQFRSAATVRITAGA 893

A.digitifera TKLEYPEQGIEITRHGVKIEQTLQKAFLGYHARHMIPTLAGIMLDMEFRAGTSAIARTTA 574

A.tenuis ----------------------------------------------------------TA 2

**DUF1943**

*

G.fascicularis NLDVH-PSLKTFWQYQSMTGNVEVKPSINTHVHAVVAIHTPFLRVGVQMQANGNSNTSLK 952

E.ancora NLNVH-PSLKNFWQYQSMTGNVEVKPSINAHVHAVVAIHTPFLRVGVQMKANGNSNTNQK 952

A.digitifera NFGVSSSSWLTFWQYNKMTGNLRITPRVNTHAHALVGIHTPLVRVGIQMKLNIRSYFDKR 634

A.tenuis NFGVTSSSWLKFWQYNIMTGNLRITPRVNTHAHALVGIHTPLVRVGIQMKLNIRSYFDKR 62

*:.* * .****: ****:.:.* :*:*.**:*.****::***:**: * .* . :

G.fascicularis IEVQAEAEKSCNFGYSIPQEQKDIIQFKAVTQGFSQQQDPQTCEQKEKQISLDLSKDHLT 1012

E.ancora VEVQAEAKKSCNFGYSIPQEQKDIFQFKAVTQGFSQQQDPQTCEQKEKQISLDLSKDHLT 1012

A.digitifera ANVVAEKGKQCSVKFNIPDRKWDVWTVKVDTEGFTQECNPDTLKTTEKAISMELNRDHIT 694

A.tenuis ANVVAEKGKQCSVKFNIPERKWDVWTVKVDTEGFTQECNPDTLKTTEKAISMELNRDHIT 122

:* ** *.*.. :.**:.: *: .*. *:**:*: :*:* : .** **::*.:**:*

G.fascicularis QLKKSCAGQALFGVQLCAEGQGPDLASLRLRQVPVFSAICQAEMRVSLAPASDSPLLIQW 1072

E.ancora QLKKSCAGKALFGVQLCAEGQGPDLPSLRLQQVPVFSAICQAEMRVSMVPATDSPPSIQW 1072

A.digitifera QLQRRCYGKDMFGVQYCVQGTFPDLTALRLQQVQLLPVIGQFDGRFTGEPAEDKPTDIEW 754

A.tenuis QLQKSCYGKDMFGVQYCVEGTFPALTALTLQQVQVLPVIGQFDGRFTGEPAEDKPTDIEW 182

**:: * *: :**** *.:* * * :* *:** :: .* * : *.: ** *.* *:*

G.fascicularis NNNIEKNDD-KEKDVSGEI--KVSSNKITRRLPYRITYKRDQQQ-LEIGSTSLVAQGYED 1128

E.ancora NNKIEKNDD-KEKDVSGEI--KASSNKTTRHLSYRISYKRNQQQ-LEIESTSLVAQGYED 1128

A.digitifera LHKHPVISKLGTQEIHGEINVLAASKAVTRKIPYKITYNRGHQRELLIKFDDLQVKGYEK 814

A.tenuis LHKHPVISKLGTQEINGEINVLAASKAVTRKIPYKITYKRGHQRELVCQVRWSSGERVRK 242

:: .. ::: *** .:*: **:: *:*:*:*.:*: * : ..

G.fascicularis AKMKLIAKGDGLRLEFGRK---GKPQYSVTVSGQIENQGKSLKVQANWTEVPEQWKTFFR 1185

E.ancora AKMKLIANGDGLRLEFGRK---EKPQYLVTVSGQIENQGRSLRVQANWTEVPEEWKTFFR 1185

A.digitifera AMMTLKVDSNGLKLNFGNKAGQKGPKYEITVSGQVENQGKCLRARARWNMLPEQWKEFLY 874

A.tenuis SDDDIESDSNGLKLDFGNEAGQKGPKYLINVSGQVENQGKCLRAQAHWNMLPEQWRDFLY 302

: : ..:**:*:**.: *:* :.****:****:.*:.:*.*. :**:*: *:

G.fascicularis MWEPQILYALQQFAWVRRTDQQTKQVALEFQLTSPMTASSTVKTPNAEAKRTKLSLPICV 1245

E.ancora RWEPQILYALQQFAWVRRTDQQTKQVALEFQLTSPMTASVTVKTPDAEAKRTKLSLPIRV 1245

A.digitifera QWEPQIWNALQQFAWVRRVNQKKKQMEFQIQLKTATTADWQLKVPNADVKRTNVRLPLRV 934

A.tenuis QWEPQIWNALQQFAWVRRVNQKKKQMEFQIQLKTATRADWELKVPNADVKRTNVLLPLRV 362

***** **********.:*:.**: :::**.: *. :*.*:*:.***:: **: *

G.fascicularis ERLPTSIQEIKDHLYAKCEVQEQSIKVFDQLRYQHNIKGGCPYVLAQEYREGKQSRFELT 1305

E.ancora ERLPTSIQEITDHLYARCEVQDQSIKVFDQLRYQHNIKGGCPYVLAQEYREGKQSRFQLT 1305

A.digitifera ERFASSYDDIMNFVFARCDVQGQNIRVFDHLQYVANIEAGCPYVLVQEHNTDRPSRIMFT 994

A.tenuis ERFASSYDDIMNFVFARCDVQGQNIRVFDQLQYVANIKRGCPYVLVQEHNTGRPSRIMFT 422

**: :* ::* :.::*:*:** *.*:***:*:* **: ******.**:. .: **: :*

G.fascicularis VKTDEQGQKTLIASFQQQ---SQQHKETVEIKP---DLTILIDGQKQTCAQQPCRSKQGD 1359

E.ancora VKIDEQGQKTLIASIQQQSQQSQQHKETVEIKP---DSTILIDGQKQTCAQQPCRSKQGD 1362

A.digitifera VQMNGKGQKTLRILIRKS-------KERVEIKANDDHPIVLIDG--QLCAKQDCQTKLQE 1045

A.tenuis VQMDGKGQKTLRILISKS-------KERVEIKANNDHPIVLIDG--QLCAKQDCQTKLQE 473

*: : :***** : :. ** **** . :**** * **:* *::* :

G.fascicularis FTVRTVQTTDGKTEIQLSTKLGLHVTMEGQRIHVYVAPFLCGRVRGLCGDADGEQWNEYK 1419

E.ancora FTVRKVQTTDGKTEIQLSTKLGLYVTMVGQRIHVYAAPSLCGRVRGLCGDADGEQWNEYK 1422

A.digitifera VTVHTAQMPGGKNQVYLHTKFGLCARIKGGQISVYASPLLQGHVRGLCGDANGEQWNEFR 1105

A.tenuis VTVHTAQMPGGK------------------------------------------------ 485

.**:..* .**

G.fascicularis DPQGRVQTLQKFIQSWQQKC 1439

E.ancora DPQGRVQTLQKFIQSWQQKC 1442

A.digitifera DRKDKIRDRQTFVKSWQQKC 1125

A.tenuis -------------------- 485

**Fig. S5.** Amino acid alignment (VG) of *A. tenuis*, other cnidarians including *Acropora digitifera* (AN5404), *Euphyllia ancora* (AGO04748.1), and *Galaxea fascicularis* (BAD74020.2)*.* Asterisks (*) indicate a perfect match among all aligned sequences, whereas dots represent conservative replacements: (.) conserved; (:) highly conserved. Domain region and motifs were indicated on the top of sequence. DUF1943; Black box with yellow highlight, the putative consensus sequence (K/RXXR) for the cleavage sites of the subtilisin family endoproteases; dotted box with green highlight.


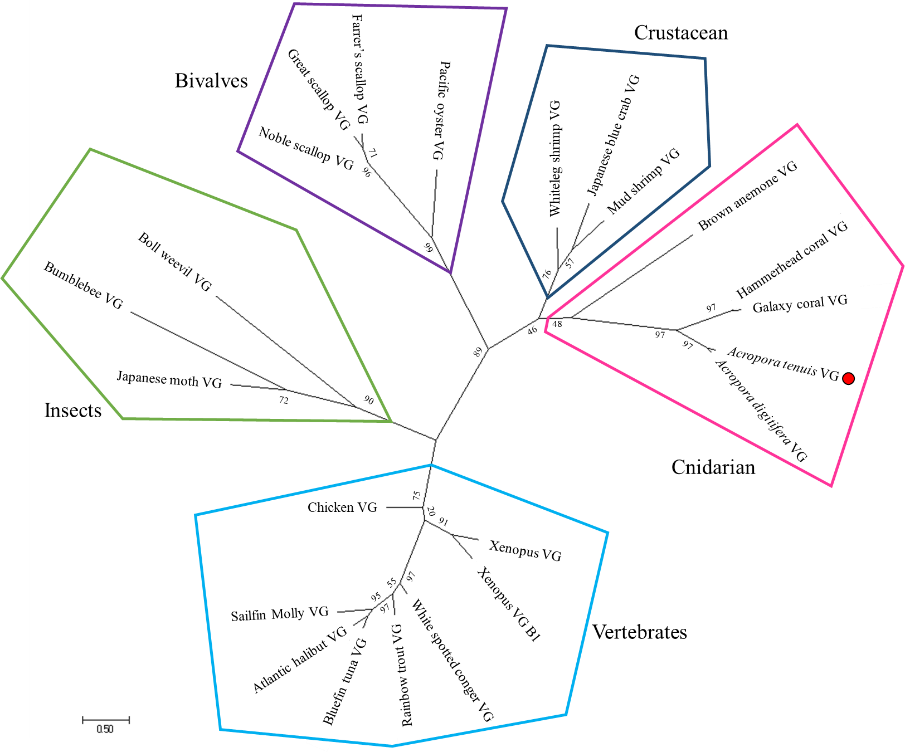


**Fig. S6.** Phylogenetic analysis comparing the amino acid sequences of Vitellogenin of various taxa. The consensus tree with 1000 bootstrap replications was obtained using the Maximum likelihood analysis with Jones-Taylor-Thornton (JTT) model. The sequences were aligned by multiple sequence alignment using MEGA7.0. Bootstrap values were expressed as percentages. Red circle express *AtVG*. The scale bar is calibrated in substitutions per site. The names and corresponding GenBank accession numbers of the proteins analysed are as follows: Brown anemone (Exaiptasia pallida) KXJ15344.1, Galaxy coral (*Galaxea fascicularis*) BAD74020.2, Hammerhead coral (*Euphyllia ancora*) AGO04748.1, *Acropora digitifera* (AN5404), *Acropora tenuis*, Atlantic halibut (*Hippoglossus hippoglossus*) ABQ58113.1, Rainbow trout (Oncorhynchus mykiss), CAA63421.1, Atlantic bluefin tuna (*Thunnus thynnus*) ACX32463.1, White sturgeon (*Acipenser transmontanus*) AAA87392.1, White spotted conger (*Conger myriaster*) BAD93275.1, Sailfin molly (*Poecilia latipinna*) ACV65040.1, Chicken (Gallus gallus) AAA49139.1, Xenopus (*Xenopus laevis*) AAA49982.1 & AC81696.1, Boll weevil (*Anthonomus grandis*) AAA27740.1, Japanese bumblebee (*Bombus hypocrita*) ACU00433.1, Japanese moth (*Saturnia japonica*) BAD91195.1, Great scallop (*Pecten maximus*) CAQ06469.2, Farrer’s scallop (*Chlamys farreri*) ADE05540, Noble scallop (*Mimachlamys nobilis*) AFO66775.1, Pacific oyster (*Crassostrea gigas*) BAC22716.1, Japanese blue crab (*Portunus trituberculatus*) AAX94762.1, Japanese mud shrimp (*Upogebia major*) BAF91417.1, Whiteleg shrimp (*Litopenaeus vannamei*), AAP76571.


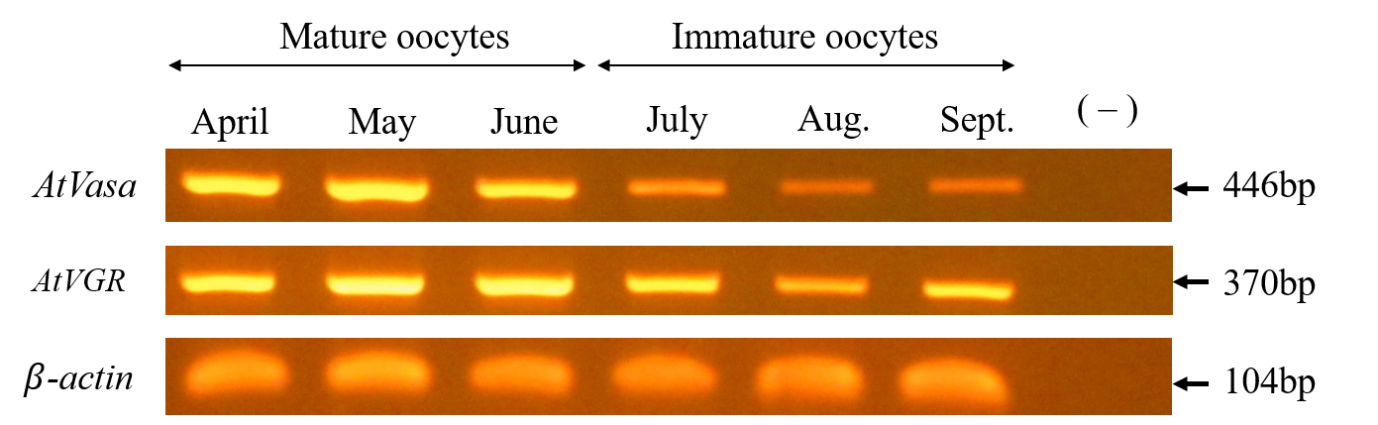


**Fig. S7**. RT-PCR analysis of *A. tenuis* Vasa and LDLR expression. Total RNA was extracted from coral branches from samples containing mature oocytes (before spawning) and samples containing immature oocytes (after spawning). The cDNA of each sample was synthesized. After amplication of *AtVasa*, *AtLDLR*, and *β-actin* using PCR, products were electrophoresed. Negative control is indicated as ( – ).


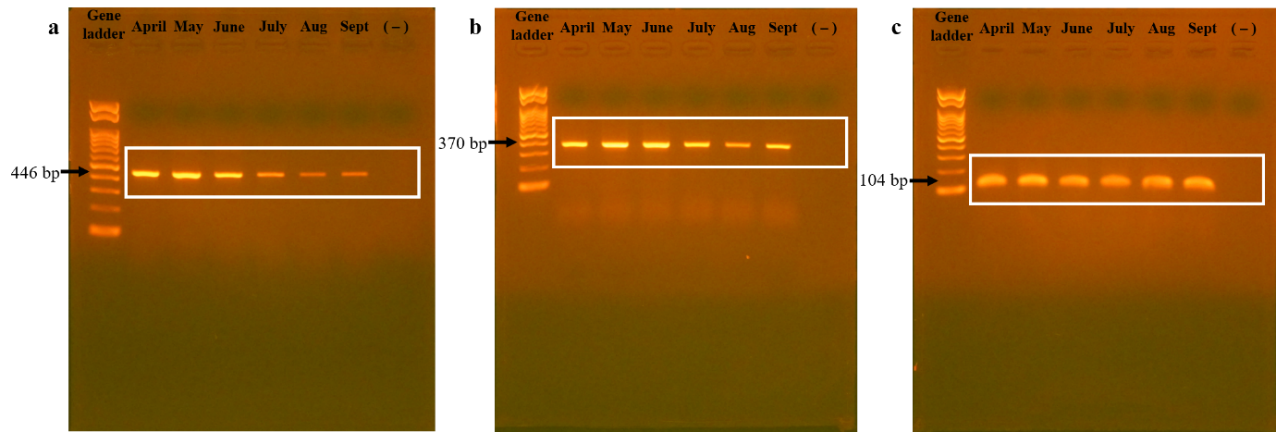


**Fig. S8**. Full-length gels of RT-PCR analysis mentioned in **Fig. S7**.

**a.** *AtVasa* ~ 446 bp; **b.** *AtLDLR* ~370 bp **c.** *β-actin* ~104bp. Position of gene ladder and coral samples from each month are labelled above their respective wells. The gene expression are shown in white rectangle box. Negative control is indicated as ( – ).


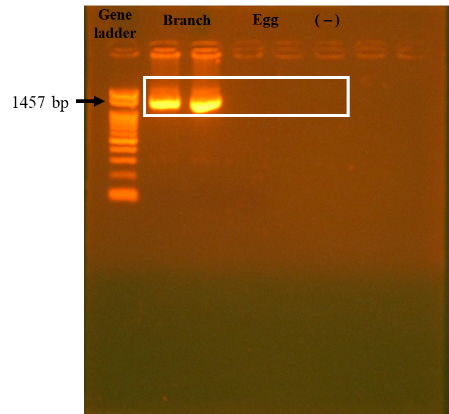


**Fig. S9**. RT-PCR analysis of *A. tenuis* VG expression. Total RNA was extracted from coral branches containing matured oocytes and eggs. The cDNA of each sample was synthesized. After amplication of *AtVG* and *β-actin* using PCR, products were electrophoresed. Negative control is indicated as ( – ).


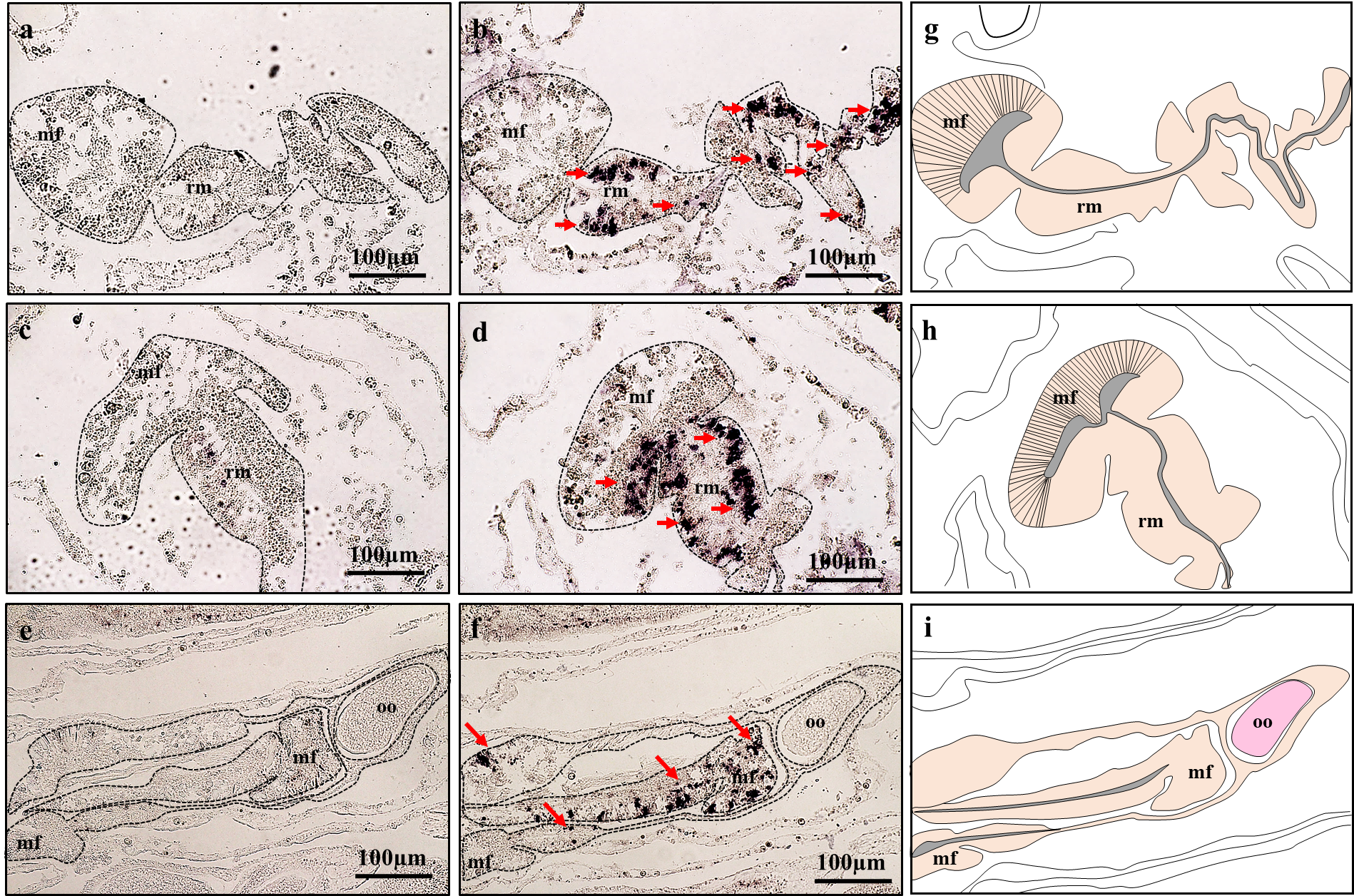


**Fig. S10**. Expression profiles of *AtVasa* transcript in mesentery tissues. **a-f**; Localization of *AtVAsa* mRNA-positive cells in the retractor muscles (rm) area of mesentery tissues. **a**, **c** and **e** showed section stained with *AtVasa* sense probe. **b, d, f** showed section stained with *AtVasa* anti-sense probe. Purple colouration (ALP reaction) indicates *AtVasa* transcript signals. **g,h,i** showed illustration of sections in **b,d,f** respectively. mf; mesenterial filament, oo; oocyte


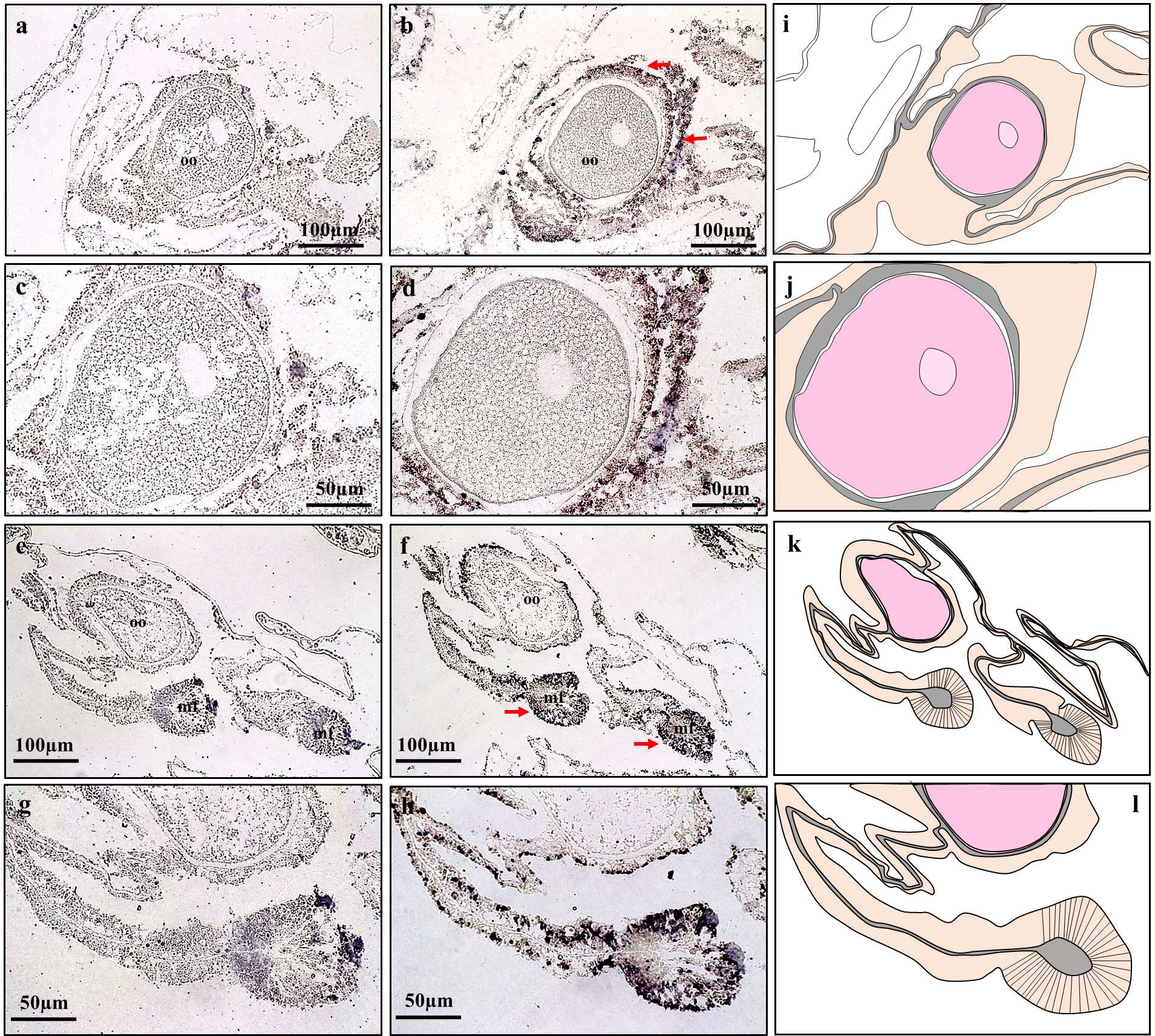


**Fig. S11**. Expression profiles of *AtLDLR* transcript in putative ovarian and mesentery tissues. **a-h**; Localization of *AtLDLR* mRNA-positive cells in putative ovarian and mesentery tissue. **a,c,e,g** showed section stained with *AtLDLR* sense probe. **b,d,f,h** showed section stained with *AtLDLR* anti-sense probe. Purple colouration (ALP reaction) indicates *AtLDLR* transcript signals. **c,d,g,h** showed higher magnification views of **a,b,e,f** respectively. **i,j,k,l** showed illustration of sections in **b,d,f,h** respectively. mf; mesenterial filament, oo; oocyte


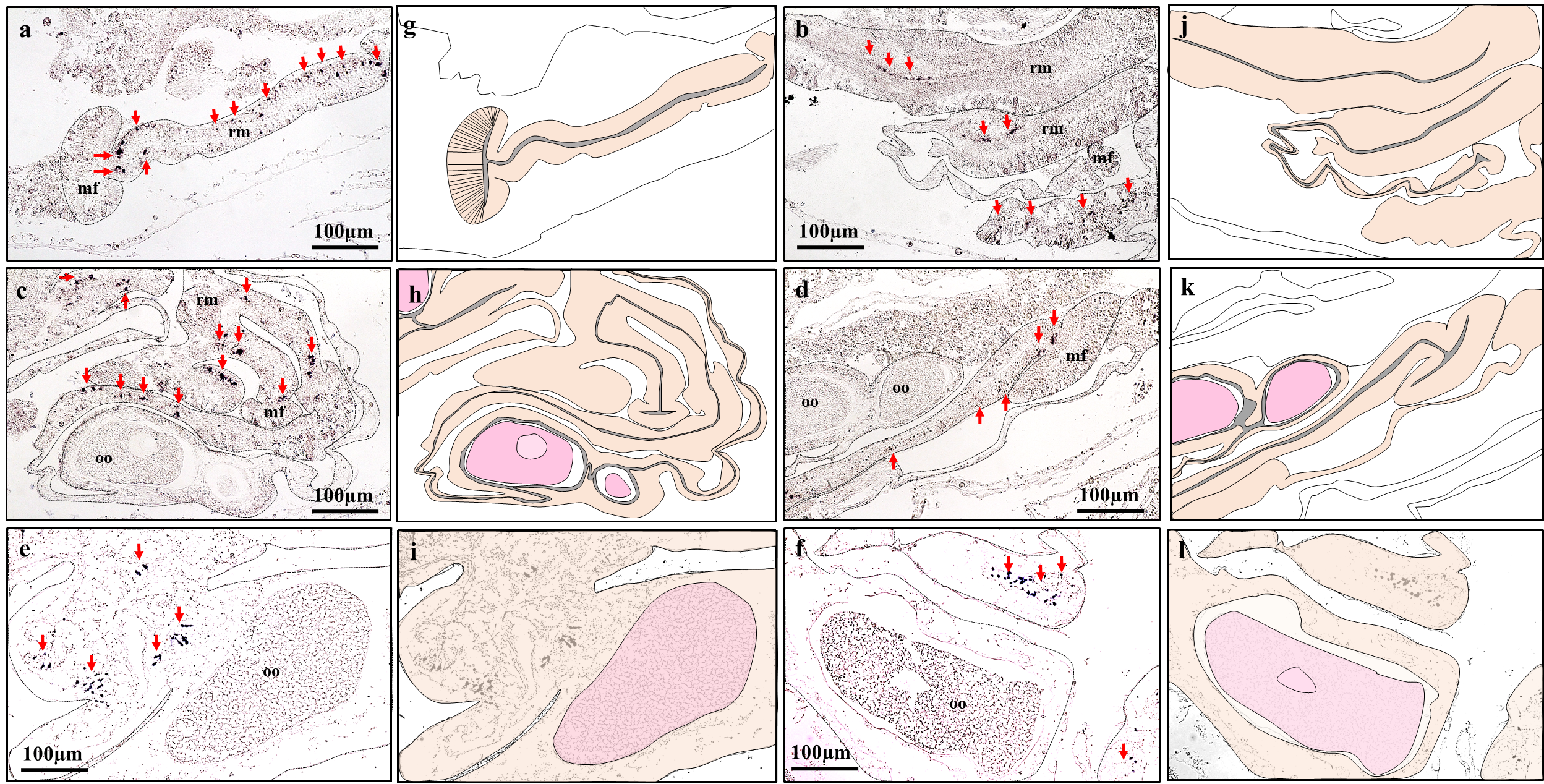


**Fig. S12.** The presence of mRNA expression *of AtVasa* in germ cells **(a-f)** and its illustration (**g-l**) in *A. tenuis* oocytes at different development stages of oogenesis. ISH: **a,b.** Stage I-II; **c,d.** Stage III; **d,e.** Stage IV-V. Arrow indicate *AtVasa* transcript signals. rm; retractor muscle, mf; mesentery filament, oo; oocyte.


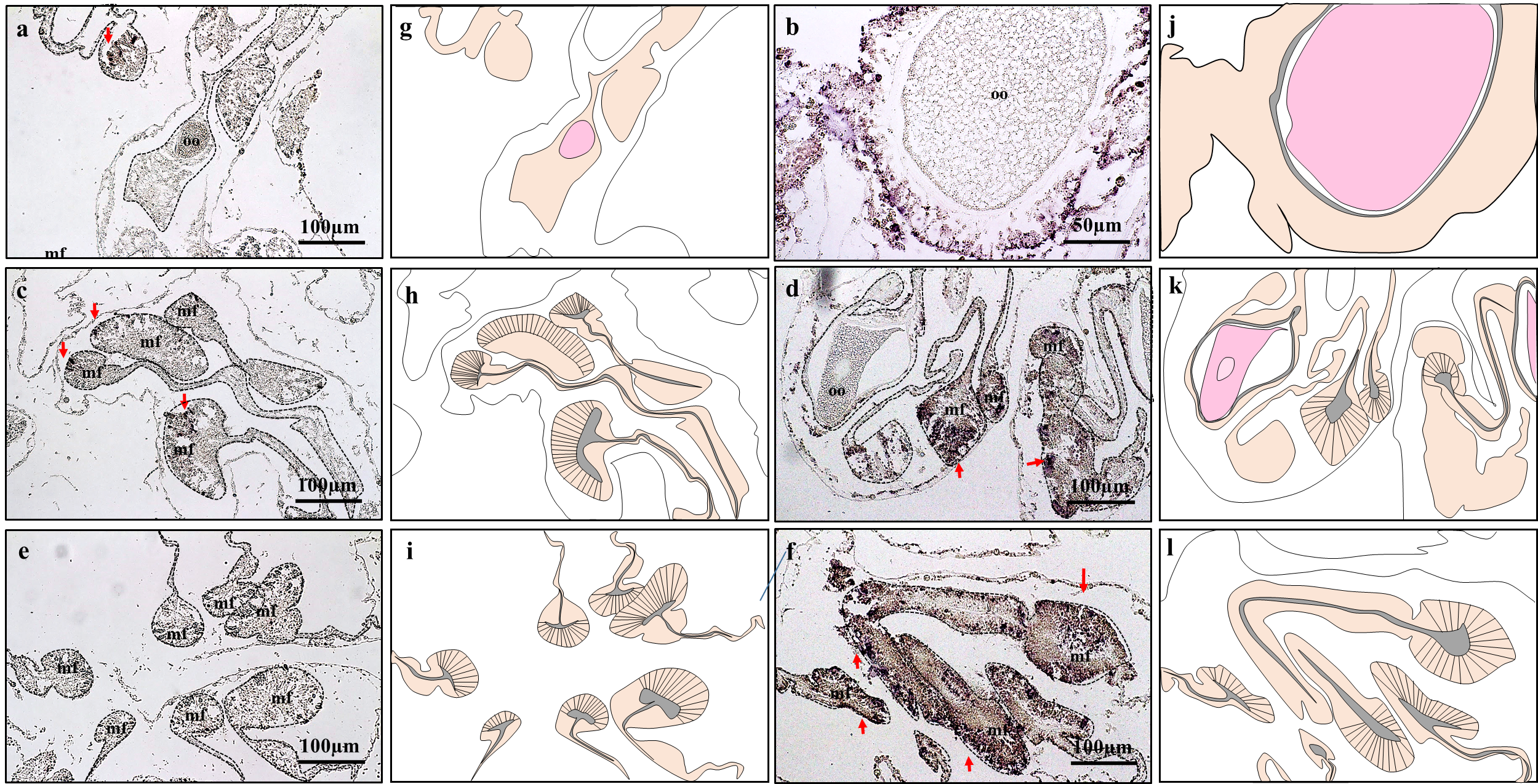


**Fig. S13**. Comparison of mRNA expression of *AtLDLR* during non-vitellogenic phase, Stage I & II (**a, c, f**) and vitellogenic phase, Stage III-V **(b, d, f)** of *A. tenuis*. Meanwhile. **g-l** showed illustration of sections in **a-f**. Arrow indicate *AtLDLR* transcript. mf; mesentery filament, oo; oocyte.


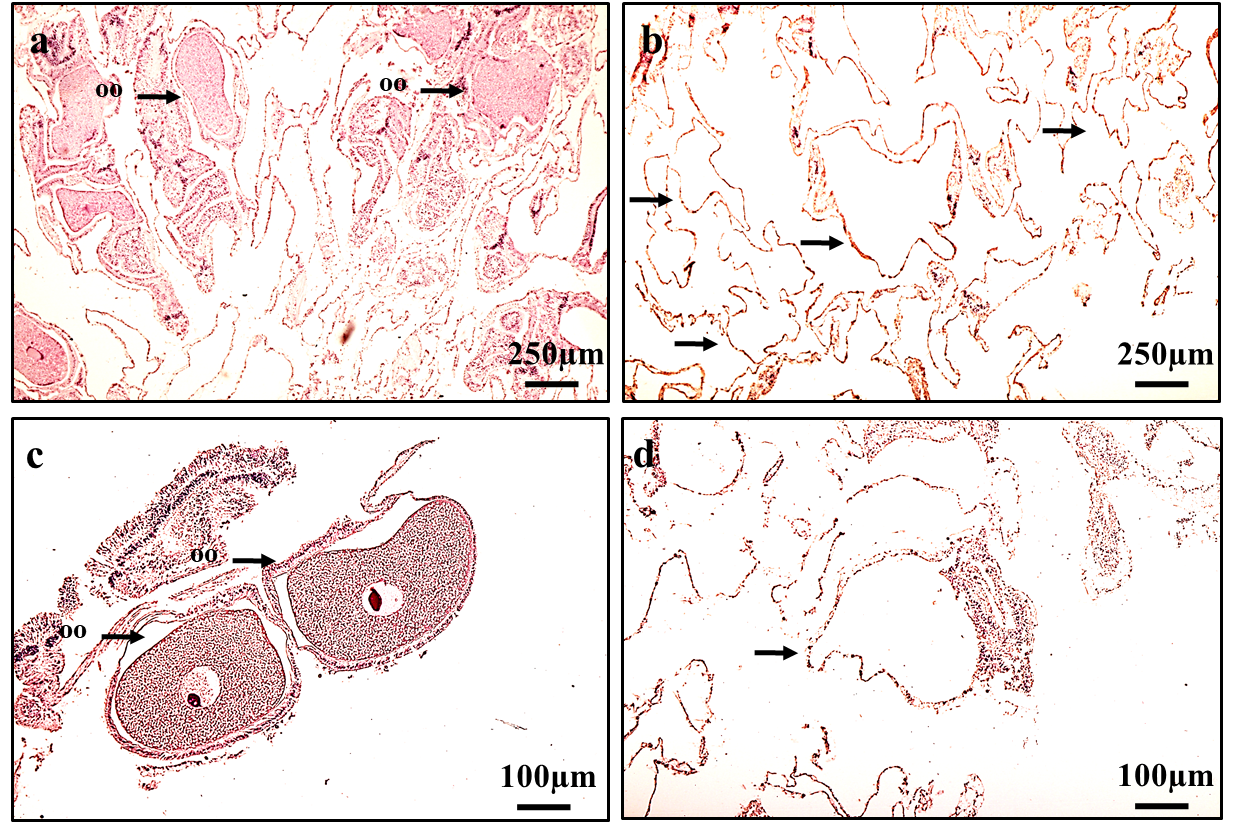


**Fig. S14**. Comparison of coral tissue sections before and after spawning takes place. **a. & c.** Mesentery with oocytes (oo). **b. & d.** Mesentery without oocyte (Post spawning stage).


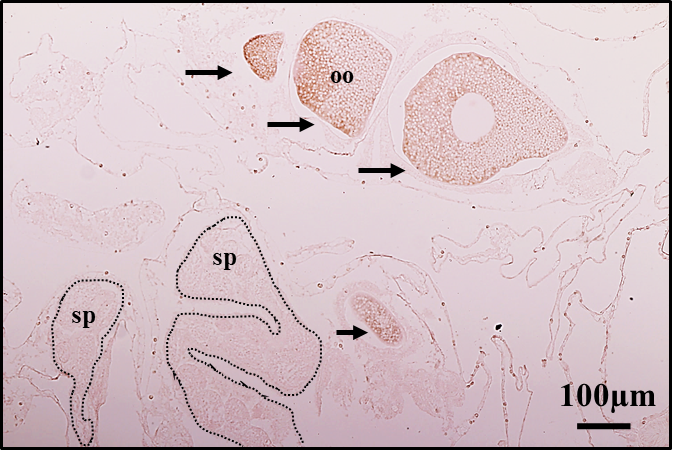


**Fig. S15**. AtVG immunoreactivity positive signal located in the cytoplasm of oocytes (oo). No AtVG immunoreactivity was observed in spermatocytes (sp) and its surrounding tissues.

**

**Fig. S16**. Western blot analysis of AtVG in coral branch tissues and eggs, as well as AtVasa in coral branch tissues. **a.** Molecular weight markers. **b.** Western blot analysis with anti-EaVg antibody. Arrow indicate AtVG immunoreactive bands of approximately 82 kDA in egg protein extract. **c.** Western blot analysis with anti-EaVas antibody. Arrow indicates AtVasa immunoreactive band of approximately 82 kDA in coral tissue protein extracts.
